# Supplementary figures and images for: Retinoic acid alleviates the reduction of Akt and Bad phosphorylation and regulates Bcl-2 family protein interactions in animal models of ischemic stroke
Source: PLoS One. 2024 May 16;19(5):e0303213. doi: 10.1371/journal.pone.0303213 (PMC11098415; doi:10.1371/journal.pone.0303213)

Figure 1D

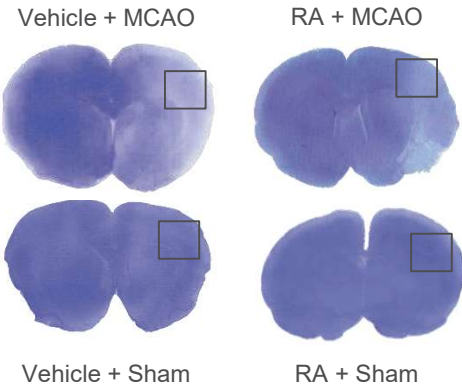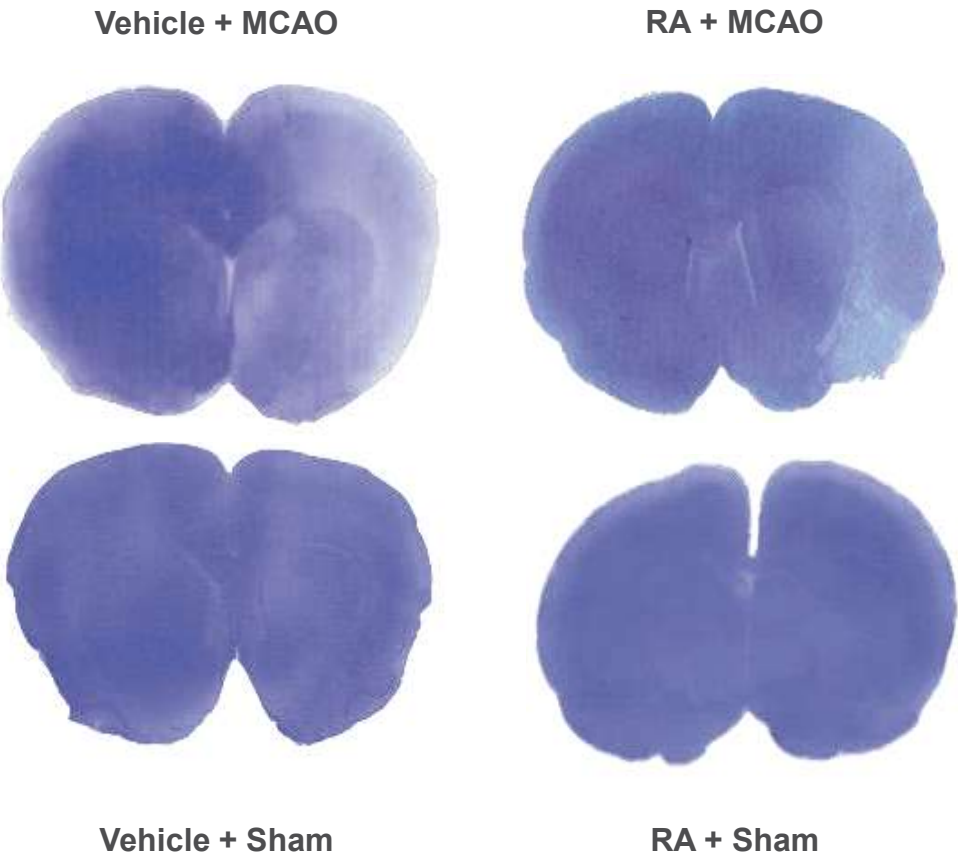

Supplement: S1 File — This is full images of Fig 1D. (PDF) [file pone.0303213.s001.pdf]

Figure 1F

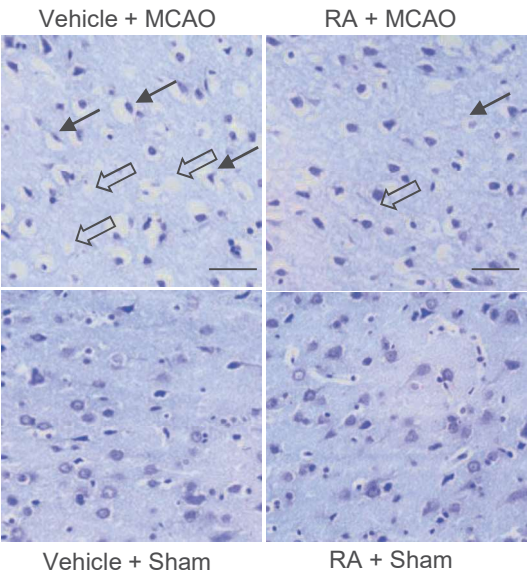

Vehicle + MCAO

RA + MCAO

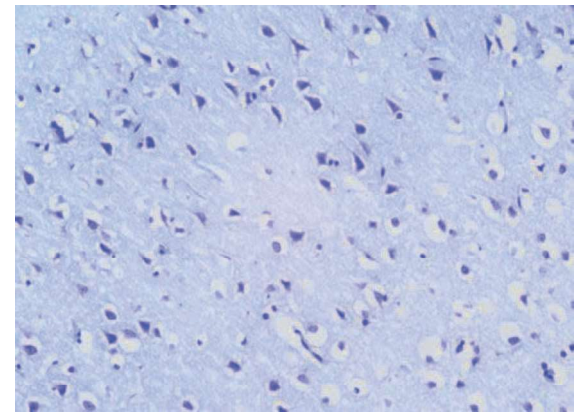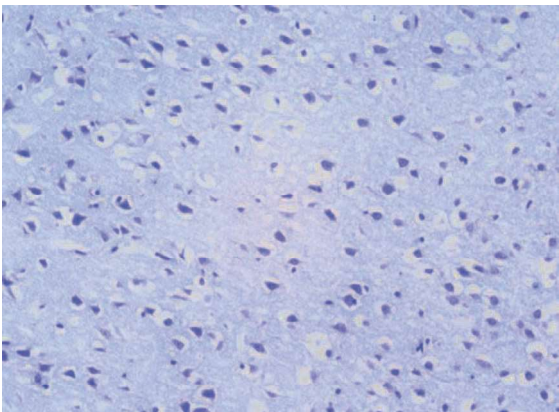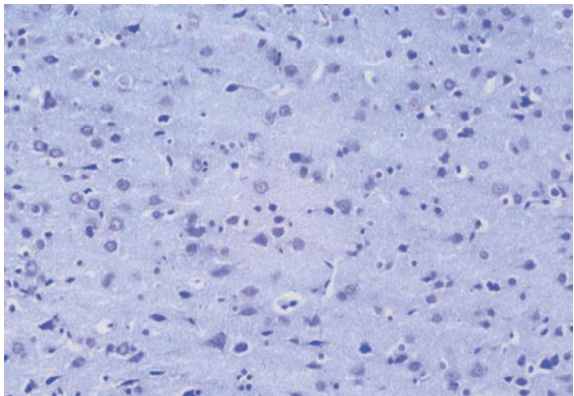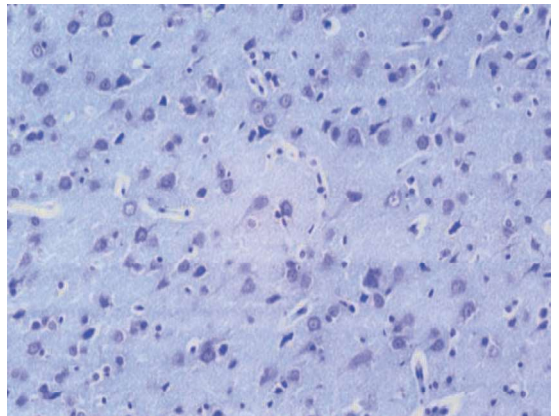

Vehicle + Sham

RA + Sham

Supplement: S2 File — Full images of Fig 1F. (PDF) [file pone.0303213.s002.pdf]

Figure 1G

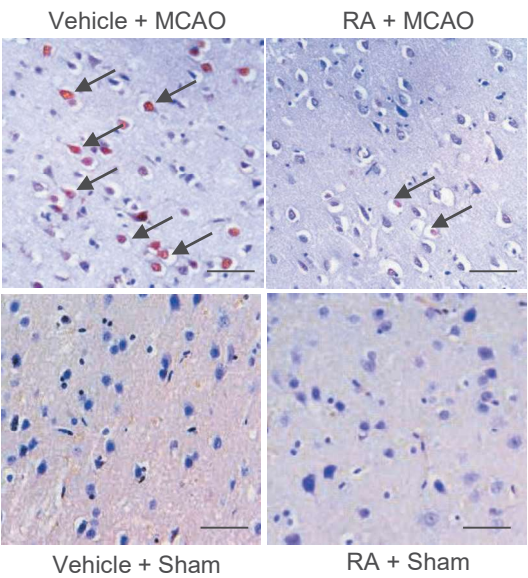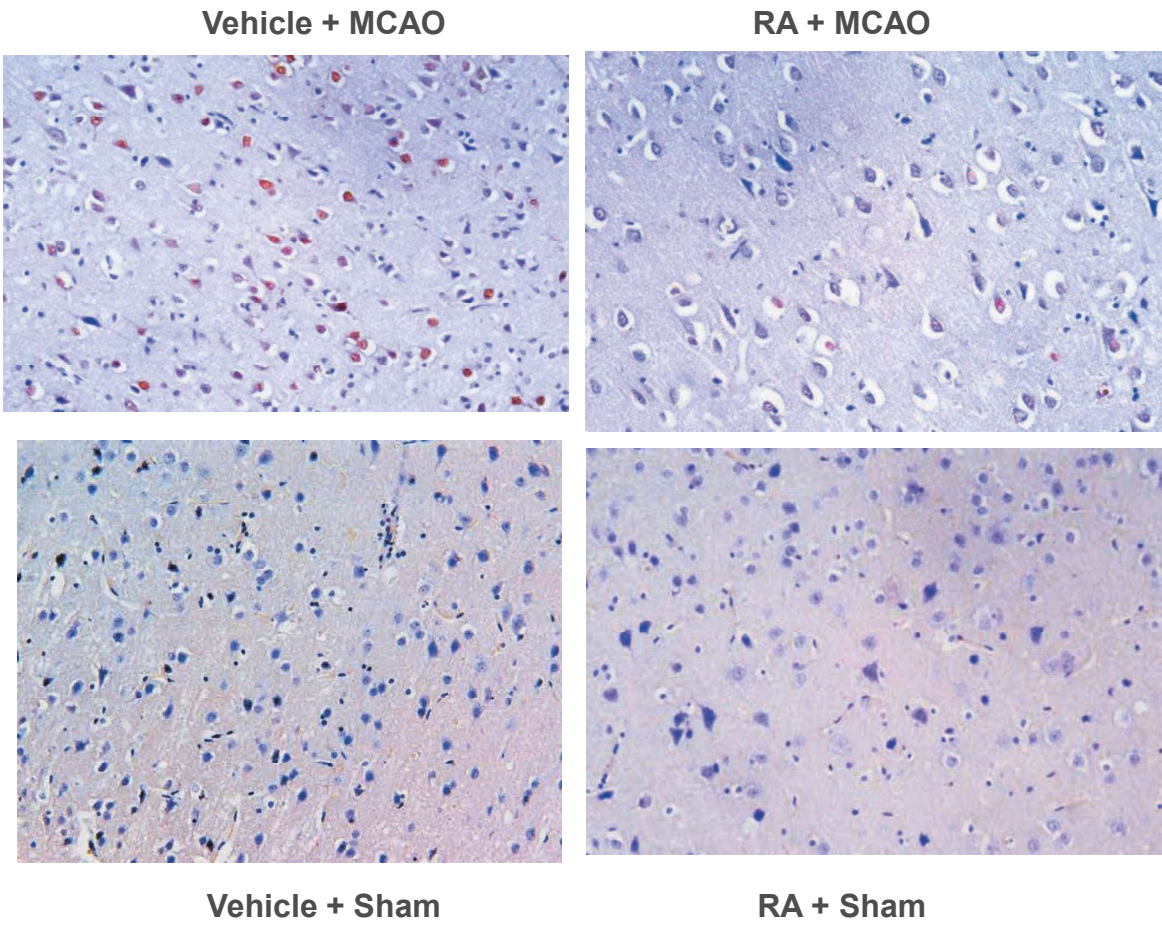

Supplement: S3 File — Full images of Fig 1G. (PDF) [file pone.0303213.s003.pdf]

Figure 2A

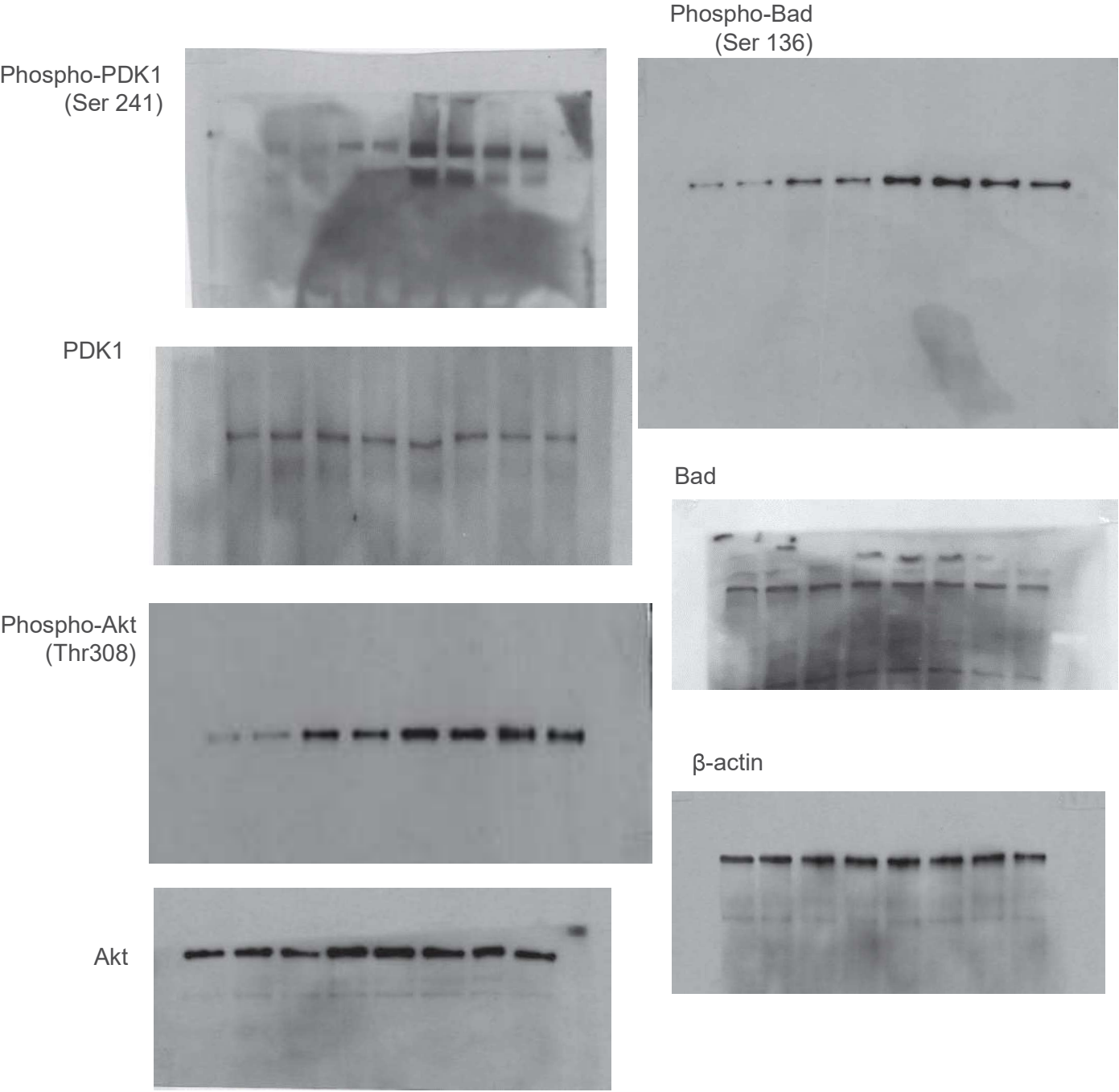

Supplement: S4 File — Full images of Fig 2A. (PDF) [file pone.0303213.s004.pdf]

Figure 3A

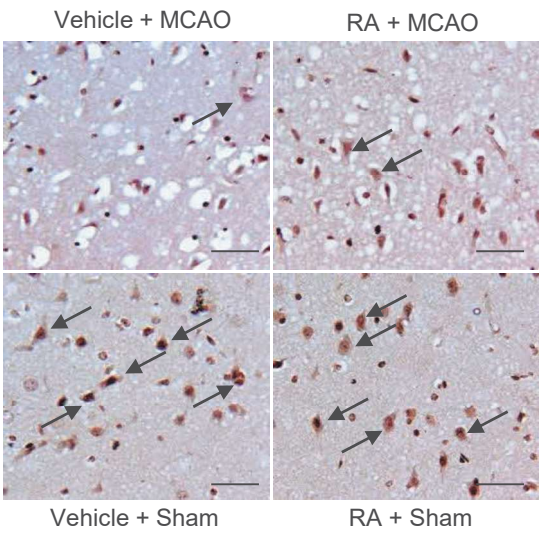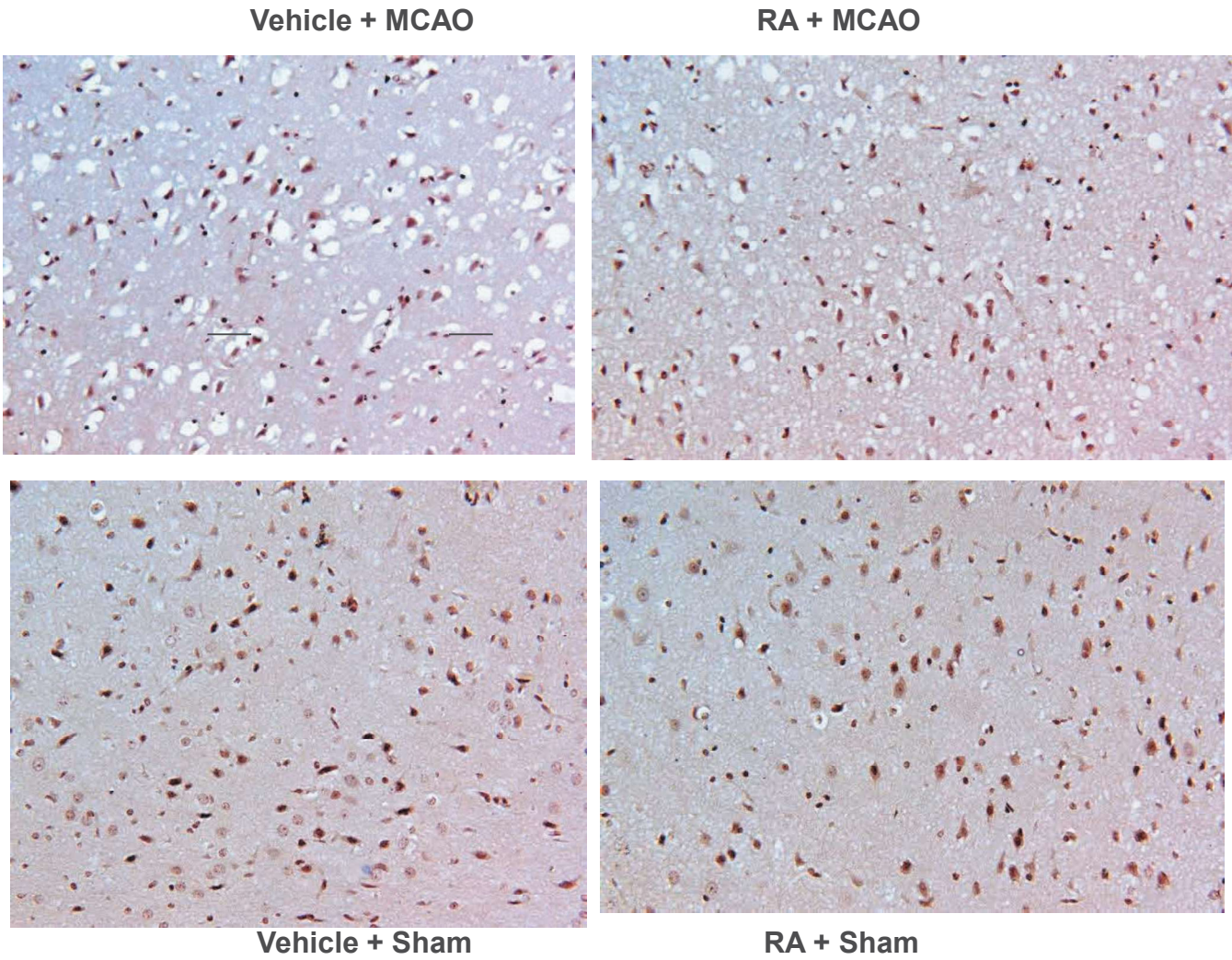

Supplement: S5 File — Full images of Fig 3A. (PDF) [file pone.0303213.s005.pdf]

Figure 3B

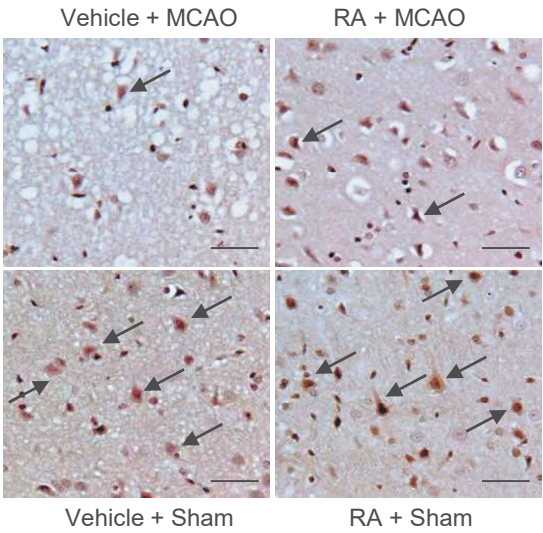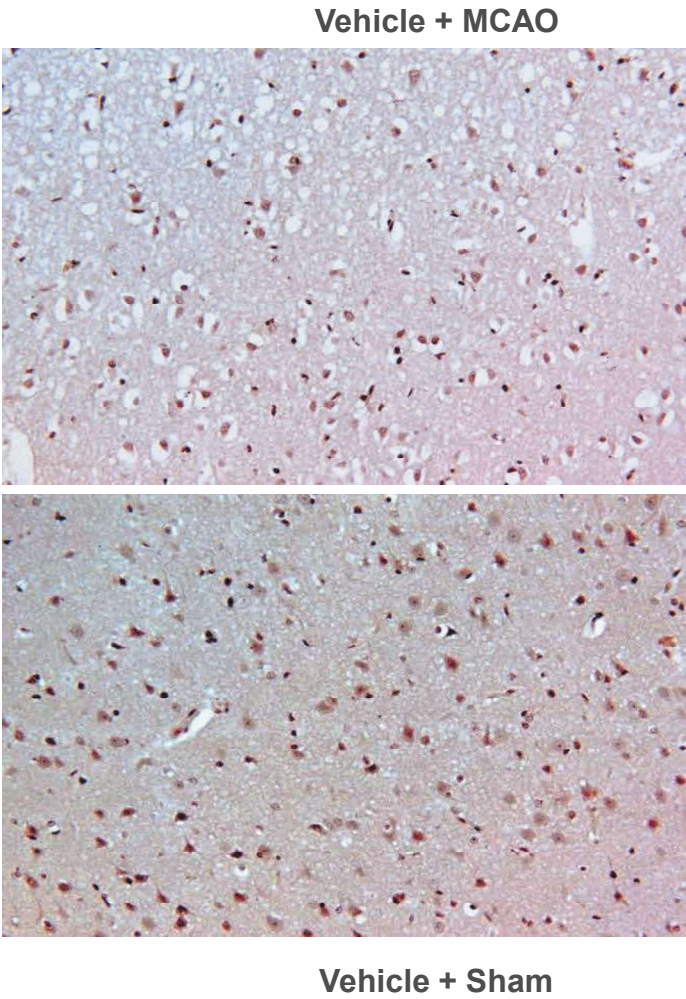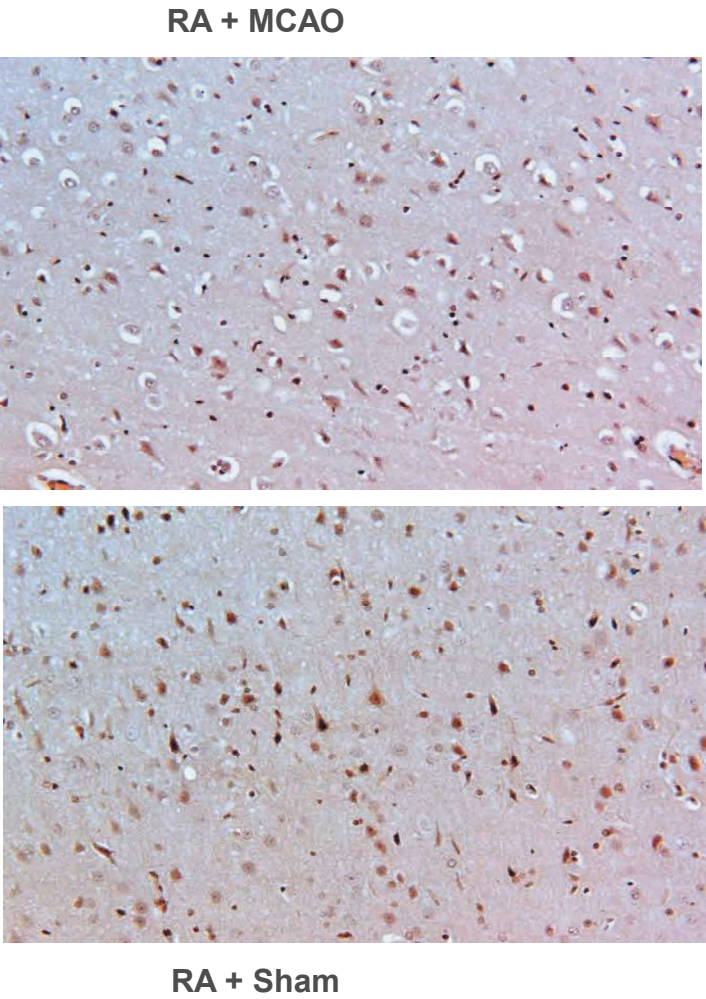

Supplement: S6 File — Full images of Fig 3B. (PDF) [file pone.0303213.s006.pdf]

Figure 4A

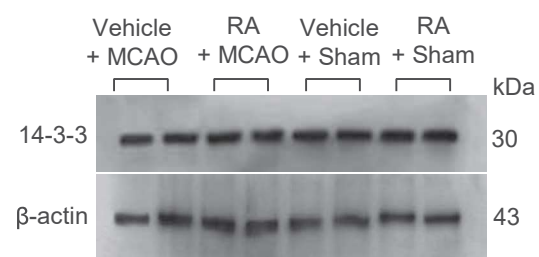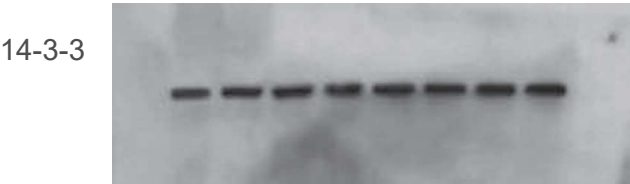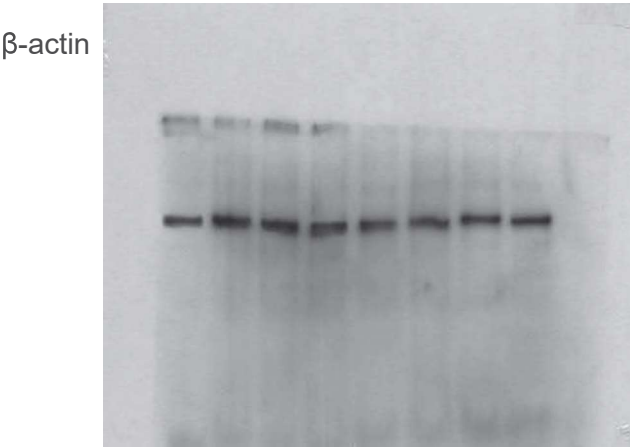

Supplement: S7 File — Full images of Fig 4A. (PDF) [file pone.0303213.s007.pdf]

Figure 4C

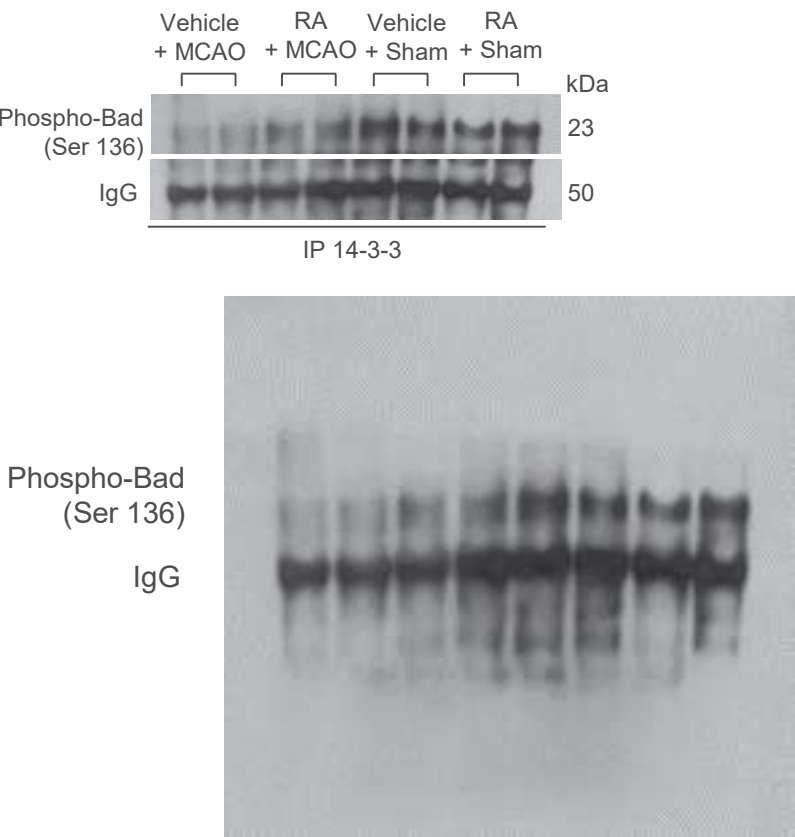

Supplement: S8 File — Full images of Fig 4C. (PDF) [file pone.0303213.s008.pdf]

Figure 5A

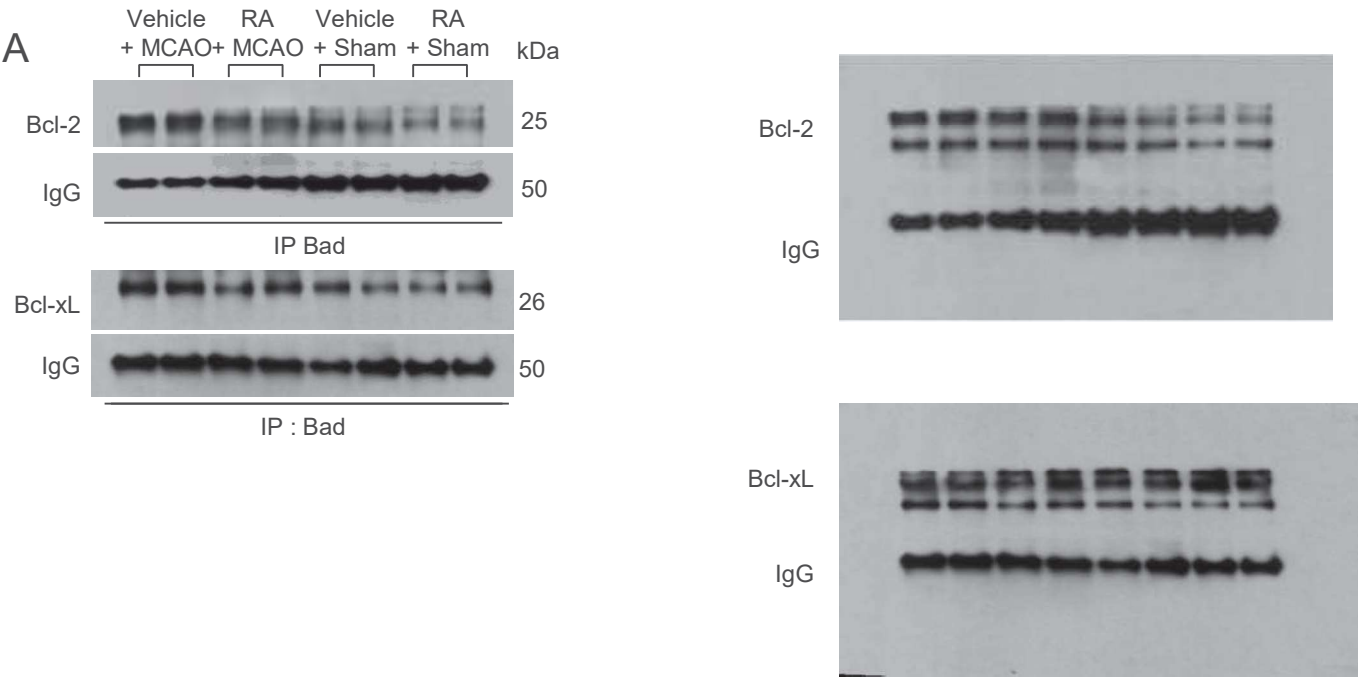

Supplement: S9 File — Full images of Fig 5A. (PDF) [file pone.0303213.s009.pdf]

Figure 5B

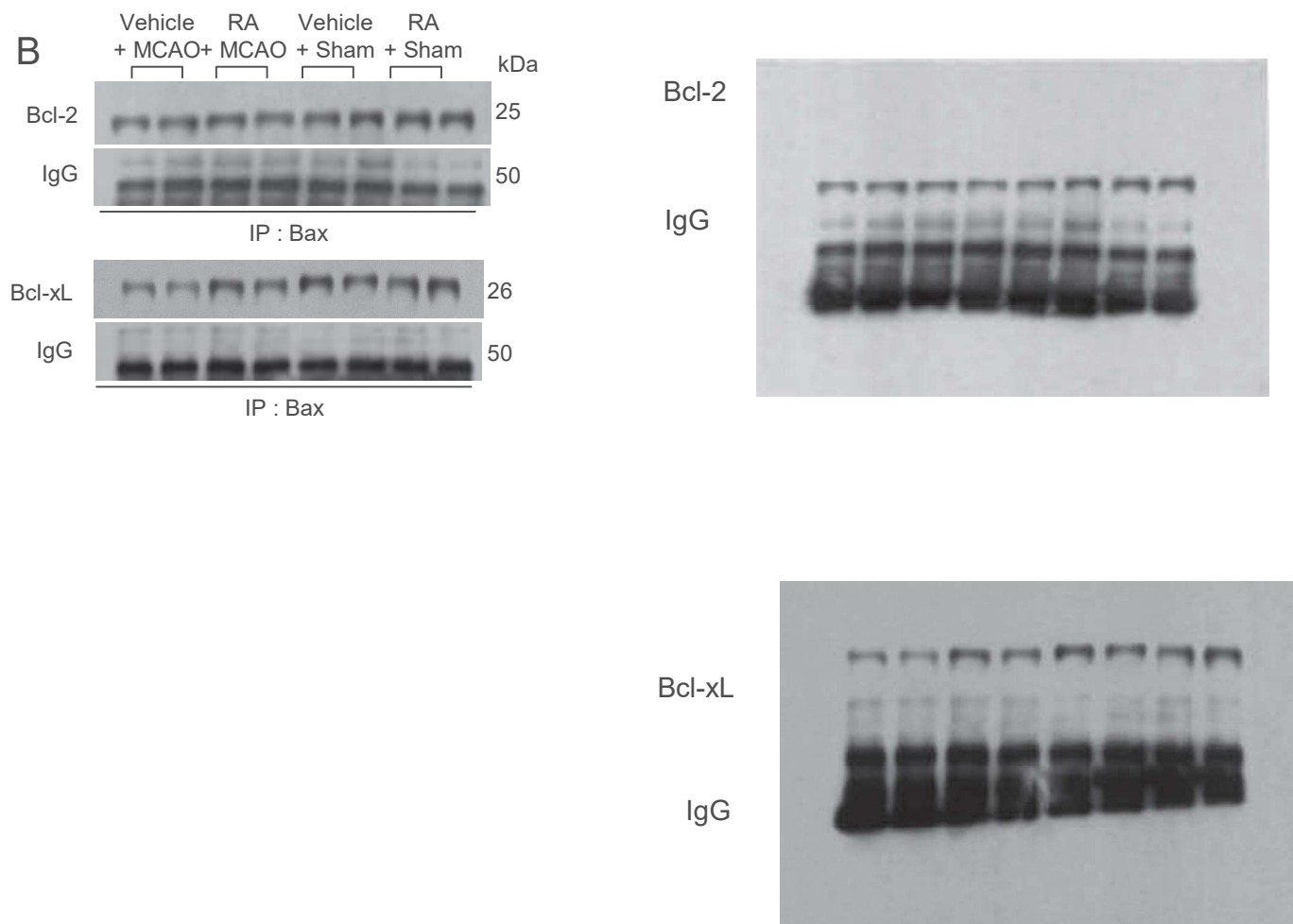

Supplement: S10 File — Full images of Fig 5B. (PDF) [file pone.0303213.s010.pdf]

Figure 6A

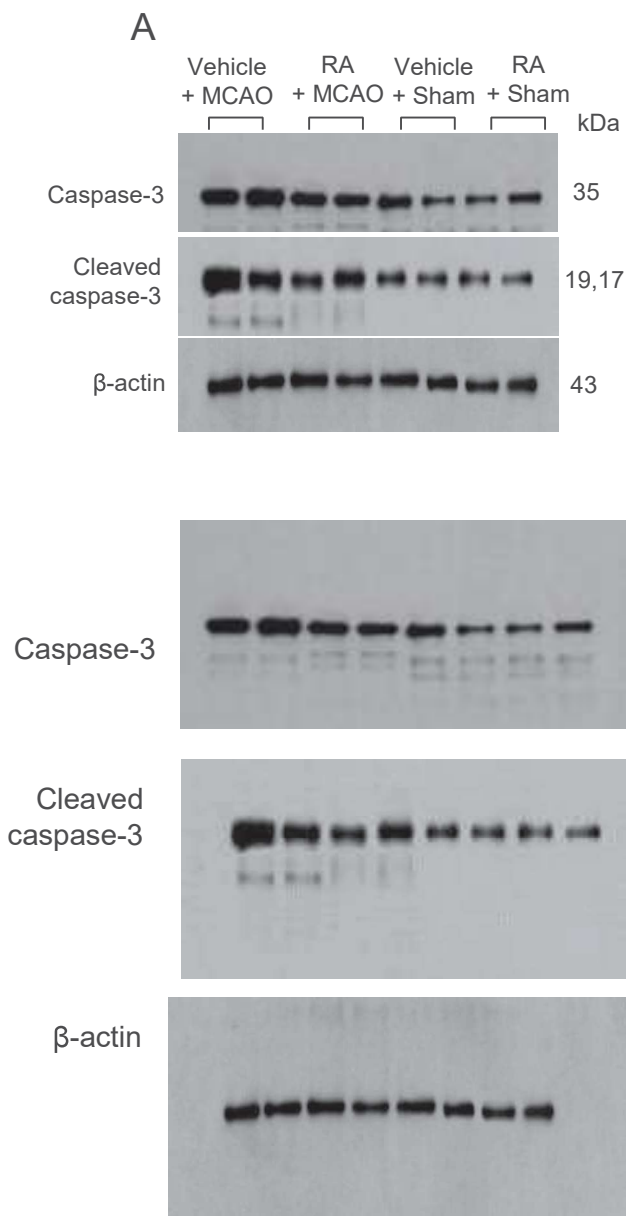

Supplement: S11 File — Full images of Fig 6A. (PDF) [file pone.0303213.s011.pdf]
