# Supplementary material for: Retinoic acid alleviates the reduction of Akt and Bad phosphorylation and regulates Bcl-2 family protein interactions in animal models of ischemic stroke
Source: PLoS One. 2024 May 16;19(5):e0303213. doi: 10.1371/journal.pone.0303213 (PMC11098415; doi:10.1371/journal.pone.0303213)
Supplement: Attachment — Submitted filename: Response to reviewer.docx [file pone.0303213.s012.docx]

**Reviewer #1:** Major Revisions

The authors showed that retinoic acid pretreatment can alleviate stroke induced by middle cerebral artery occlusion. They showed this by neurological scoring and other tests with histopathological changes and western blot analysis for protein expressions.

1. The author fails to show a direct mechanism in which retinoic acid is exerting the effect observed.

**Response:**

We appreciate for your kind comments. We tried to answer your questions very carefully.

Retinoic acid has several properties such as anti-oxidation (Ahlemeyer., 2001), anti-inflammation (Oliveira et al., 2018), anti-cancer (Hunsu et at., 2021). Especially in the brain, retinoic acid has neuroprotective effects in neurodegenerative diseases such as amyotrophic lateral sclerosis (Zhu et al., 2020), Parkinson's disease (Esteves et al., 2015) and ischemic stroke (Cai et al., 2019). In this study, we focused on the specific protective mechanism of retinoic acid on cerebral ischemia. The signal pathways involved in stroke is very diverse and complex. The regulatory pathways of retinoic acid in ischemic strokes are also diverse and complex. Among these multiple pathways, we focused on the PI3K/Akt pathway, which is a signal transduction pathway that promotes survival and growth in response to extracellular signals. Akt pathway regulates glucose metabolism, protein synthesis, mitochondrial metabolism, lipid metabolism, angiogenesis, autophagy, proliferation, and cell growth (Avan et al., 2016). It is a representative pathway related to cell survival. The regulation of Akt pathway by retinoic acid in stroke has not been reported much. Akt inhibits apoptosis through phosphorylation of proapoptotic proteins such as Bad and forkhead transcription factor (FKHR). However, dephosphorylated Bad forms heterodimers with Bcl-2 and Bcl-xL to inactivate them, allowing apoptosis. We showed that retinoic acid treatment alleviates MCAO-induced decreases in phospho-PDK1, phospho-Akt and phospho-Bad expression. Retinoic acid attenuated decrease in interaction between phospho-Bad and 14-3-3 caused by MCAO damage. Furthermore, retinoic acid mitigated the increase in Bcl-2/Bad and Bcl-xL/Bad binding levels and the reduction in Bcl-2/Bax and Bcl-xL/Bax binding levels caused by MCAO damage. Retinoic acid alleviated MCAO-induced increase of caspase-3 and cleaved caspase-3 expression. We demonstrate that retinoic acid prevented apoptosis against cerebral ischemia through phosphorylation of Akt and Bad, maintenance of phospho-Bad and 14-3-3 binding, and regulation of Bcl-2 family protein interactions. Retinoic acid exerts neuroprotective effects through various protective mechanisms during brain injury. However, it is difficult to discuss all protective mechanism of retinoic acid in this study. Among these various mechanisms, we focused on the PI3K/Akt signal pathway, which is a representative survival pathway. Previous studies demonstrated that retinoic acid activated the Akt signaling pathway and promoted cell survival in various tissues. However, data on the neuroprotective effects of retinoic acid on the PI3K-Akt signaling pathway in cerebral ischemia are limited. It has not been previously reported whether retinoic acid regulates the expression of phospho-Bad and the interaction between phospho-Bad and 14-3-3 in a stroke animal model. Therefore, we focused on the regulatory mechanism of Akt and its downstream target Bad by retinoic acid in cerebral ischemia. We regret that this study did not show various neuroprotective mechanism of retinoic acid in stroke animal model. However, we clearly showed that retinoic acid is involved in neuroprotection through the regulation of Akt and its downstream targets Bad in ischemic brain injury. We suggest that various protective mechanisms of retinoic acid should be researched. Thus, we plan to study other mechanisms in future research with reference to your kind comments.

**References**

Ahlemeyer B, Bauerbach E, Plath M, Steuber M, Heers C, Tegtmeier F, et al. Retinoic acid reduces apoptosis and oxidative stress by preservation of SOD protein level. Free Radic Biol Med. 2001;30(10):1067-77.

Oliveira LM, Teixeira FME, Sato MN. Impact of retinoic acid on immune cells and inflammatory diseases. Mediators Inflamm. 2018;2018:3067126.

Hunsu VO, Facey COB, Fields JZ, Boman BM. Retinoids as chemo-preventive and molecular-targeted anti-cancer therapies. International Journal of Molecular Sciences. 2021; 22(14):7731.

Zhu Y, Liu Y, Yang F, Chen W, Jiang J, He P, et al. All-trans retinoic acid exerts neuroprotective effects in amyotrophic lateral sclerosis-like Tg (SOD1*G93A)1Gur mice. Mol Neurobiol. 2020;57(8):3603-3615.

Esteves M, Cristóvão AC, Saraiva T, Rocha SM, Baltazar G, Ferreira L, et al. Retinoic acid-loaded polymeric nanoparticles induce neuroprotection in a mouse model for Parkinson's disease. Front Aging Neurosci. 2015;7:20.

Cai W, Wang J, Hu M, Chen X, Lu Z, Bellanti JA, et al. All trans-retinoic acid protects against acute ischemic stroke by modulating neutrophil functions through STAT1 signaling. J Neuroinflammation. 2019;16(1):175.

Avan A, Narayan R, Giovannetti E, Peters GJ. Role of Akt signaling in resistance to DNA-targeted therapy. World J Clin Oncol. 2016;7(5):352-369.

We added a sentence to the discussion section to make it easier to understand.

Retinoic acid exerts neuroprotective effects through various protective mechanisms during brain injury. However, it is difficult to discuss all protective mechanism of retinoic acid in this study. We focused on the regulatory mechanism of Akt and its downstream target Bad by retinoic acid in cerebral ischemia. We clearly showed that retinoic acid is involved in neuroprotection through the regulation of Akt and Bad in ischemic brain injury. We suggest that various protective mechanisms of retinoic acid should be researched.

2. The signaling pathway observed is not the only major pathway involved in stroke, other pathways have been implicated as well. Moreso, retinoic acid not only activates Akt/PI3K pathway alone. The changes in other stroke pathways in relation to retinoic acid should also be investigated.

**Response:**

We appreciate for your kind comments

Ischemic stroke is caused primarily by an interruption of cerebral blood flow, causes severe nerve damage, and is one of the leading causes of death and disability worldwide. It induces cell excitotoxicity, mitochondrial dysfunction, neuroinflammation, blood-brain barrier damage, and apoptotic processes. It is also known that the mechanism of causing ischemic stroke is very complicated. Moreover, the signaling pathway of ischemic stroke is very diverse and complex. The typical signal pathway involved in stroke is as follows; phosphatidylinositol 3-kinase (PI3K)-Akt signaling pathway, phosphatase and tensin homolog (PTEN) signaling pathway, death-associated protein kinase 1 (DAPK1) signaling pathway, postsynaptic density protein-95 (PSD95)/neuronal nitric oxide synthase (nNOS) signaling pathways, hypoxia-inducible factor (HIF) signaling pathway, nuclear factor E2-related factor 2 (Nrf2) signaling pathway, casein kinase 2 (CK2) signaling pathway, mTOR-related signaling pathways, and p53-mediated apoptotic pathway (Jo et al., 2012, Zhang et al., 2013, Shamloo et al., 2005, Guo et al., 2009, Dinkova-Kostova and Abramov, 2015, Bastian et al., 2019, Perez-Alvarez et al., 2018, Leker et al., 2004). As mentioned above, stroke is associated with various signaling pathways, but it is difficult to discuss all pathways in this study. Among these various signaling pathways, we focused on the PI3K/Akt signal pathway, which is a representative survival pathway. We investigated the change of Akt and its downstream target Bad by retinoic acid in cerebral ischemia.

Reference

Jo H, Mondal S, Tan D, Nagata E, Takizawa S, Sharma AK, et al. Small molecule-induced cytosolic activation of protein kinase Akt rescues ischemia-elicited neuronal death. Proc Natl Acad Sci USA. 2012;109: 10581-10586.

Zhang S, Taghibiglou C, Girling K, Dong Z, Lin SZ, Lee W, et al. Critical role of increased PTEN nuclear translocation in excitotoxic and ischemic neuronal injuries. J Neurosci. 2013;33: 7997-8008.

Shamloo M, Soriano L, Wieloch T, Nikolich K, Urfer R, Oksenberg D. Death-associated protein kinase is activated by dephosphorylation in response to cerebral ischemia. J Biol Chem. 2005;280: 42290-4229.

Guo S, Miyake M, Liu KJ, Shi H. Specific inhibition of hypoxia inducible factor 1 exaggerates cell injury induced by in vitro ischemia through deteriorating cellular redox environment. J Neurochem. 209;108: 1309-1321.

Dinkova-Kostova AT, Abramov AY. The emerging role of Nrf2 in mitochondrial function. Free Radic Biol Med. 2015;88: 179-188.

Bastian C, Quinn J, Tripathi A, Aquila D, McCray A, Dutta R, et al. CK2 inhibition confers functional protection to young and aging axons against ischemia by differentially regulating the CDK5 and AKT signaling pathways. Neurobiol Dis. 2019;126: 47-61.

Perez-Alvarez MJ, Villa Gonzalez M, Benito-Cuesta I, Wandosell FG. Role of mTORC1 controlling proteostasis after brain ischemia. Front Neurosci. 2018;12: 60.

Leker RR, Aharonowiz M, Greig NH, Ovadia H. The role of p53-induced apoptosis in cerebral ischemia: effects of the p53 inhibitor pifithrin alpha. Exp Neurol. 2004;187: 478-486.

In addition, retinoic acid plays a variety of roles including anti-oxidation, anti-inflammation, and anti-cancer., Retinoic acid inhibited cell death through regulation of apoptosis-related proteins (Kang et al., 2021). It regulates calcium concentration through the regulation of calcium binding proteins and inhibits apoptosis in response to brain ischemic injury (Kang et al., 2023). It also modulated MAP kinase pathway and caspase cascade. In previous studies, retinoic acid activated the Akt signaling pathway and promoted cell survival in various tissues. However, data on the neuroprotective effects of retinoic acid on the PI3K-Akt signaling pathway in cerebral ischemia are limited. It has not been previously reported whether retinoic acid regulates the expression of phospho-Bad and the interaction between phospho-Bad and 14-3-3 in a stroke animal model. Therefore, this study was designed to investigate the neuroprotective effects of retinoic acid on cerebral ischemia and the regulation of phospho-Akt and phospho-Bad by retinoic acid. In addition, we examined whether retinoic acid inhibits apoptosis and protects brain tissues from cerebral ischemia by controlling the binding of phospho-Bad with 14-3-3 and binding of Bcl-2 and Bcl-xL to Bad or Bax. Therefore, we focused on the regulatory mechanism of AKT and its downstream target Bad by retinoic acid in cerebral ischemia.

We added sentences to introduction section to make it easier to understand.

Ischemic stroke induces cell excitotoxicity, mitochondrial dysfunction, blood-brain barrier damage, neuroinflammation, and apoptotic processes. It is also known that the mechanism of causing ischemic stroke is very complicated. Moreover, the signaling pathway of ischemic stroke is very diverse and complex. The signal pathways involved in stroke are PI3K/Akt signaling pathway, phosphatase and tensin homolog signaling pathway, death-associated protein kinase 1 signaling pathway, neuronal nitric oxide synthase signaling pathways, hypoxia-inducible factor signaling pathway, nuclear factor E2-related factor 2 signaling pathway, casein kinase 2 signaling pathway, mTOR-related signaling pathways, and p53-mediated apoptotic pathway [23-30]. As mentioned above, stroke is associated with various signaling pathways, but it is difficult to discuss all pathways in this study. Among these various signaling pathways, we focused on the PI3K/Akt signal pathway, which is a representative survival pathway

We added references to reference list.

23. Jo H, Mondal S, Tan D, Nagata E, Takizawa S, Sharma AK, et al. Small molecule-induced cytosolic activation of protein kinase Akt rescues ischemia-elicited neuronal death. Proc Natl Acad Sci USA. 2012;109: 10581-10586.

24. Zhang S, Taghibiglou C, Girling K, Dong Z, Lin SZ, Lee W, et al. Critical role of increased PTEN nuclear translocation in excitotoxic and ischemic neuronal injuries. J Neurosci. 2013;33: 7997-8008.

25. Shamloo M, Soriano L, Wieloch T, Nikolich K, Urfer R, Oksenberg D. Death-associated protein kinase is activated by dephosphorylation in response to cerebral ischemia. J Biol Chem. 2005;280: 42290-4229.

26. Guo S, Miyake M, Liu KJ, Shi H. Specific inhibition of hypoxia inducible factor 1 exaggerates cell injury induced by in vitro ischemia through deteriorating cellular redox environment. J Neurochem. 209;108: 1309-1321.

27. Dinkova-Kostova AT, Abramov AY. The emerging role of Nrf2 in mitochondrial function. Free Radic Biol Med. 2015;88: 179-188.

28. Bastian C, Quinn J, Tripathi A, Aquila D, McCray A, Dutta R, et al. CK2 inhibition confers functional protection to young and aging axons against ischemia by differentially regulating the CDK5 and AKT signaling pathways. Neurobiol Dis. 2019;126: 47-61.

29. Perez-Alvarez MJ, Villa Gonzalez M, Benito-Cuesta I, Wandosell FG. Role of mTORC1 controlling proteostasis after brain ischemia. Front Neurosci. 2018;12: 60.

30. Leker RR, Aharonowiz M, Greig NH, Ovadia H. The role of p53-induced apoptosis in cerebral ischemia: effects of the p53 inhibitor pifithrin alpha. Exp Neurol. 2004;187: 478-486.

Minor revisions

1. Duration of the occlusion not mentioned.

Middle cerebral artery was occluded for 24 hours.

We mentioned the occlusion period in the following sentences.

Animals were assessed on neurobehavioral tests 24 h after surgery and brain tissues were collected for further study.”

We added the sentence to make it easier to understand.

Middle cerebral artery was occluded for 24 h.

2. Interval between the surgery and testing if 24 hours is short.

**Response:**

Thank you for your kind review. Animal models of MCAO were used to induce cerebral ischemia, and the middle cerebral artery was blocked for 24 hours. This surgical method has been used in previous studies. We have previously reported the damage of cerebral cortex due to MCA occlusion for 24 hours. We think that this experimental model is suitable for the study of the neuroprotective effects of retinoic acid. We have already reported several papers demonstrating the neuroprotective effects of retinoic acid using this experimental model (Kang et al., 2021; 2022a; 2022b; 2023a;2023b)

Kang JB, Park DJ, Shah MA, Koh PO. Retinoic acid exerts neuroprotective effects against focal cerebral ischemia by preventing apoptotic cell death. Neurosci Lett. 2021. 757:135979.

Kang JB, Koh PO. Identification of changed proteins by retinoic acid in cerebral ischemic damage: a proteomic study. J Vet Med Sci. 2022a. 84(9):1194-1204.

Kang JB, Shah MA, Park DJ, Koh PO. Retinoic acid regulates the ubiquitin-proteasome system in a middle cerebral artery occlusion animal model. Lab Anim Res. 2022b. 38(1):13.

Kang JB, Koh PO. Retinoic Acid Has Neuroprotective effects by modulating thioredoxin in ischemic brain damage and glutamate-exposed neurons. Neuroscience. 2023a. 521:166-181.

Kang JB, Park DJ, Koh PO. Retinoic acid prevents the neuronal damage through the regulation of parvalbumin in an ischemic stroke model. Neurochem Res. 2023b. 48(2):487-501.

We cited the references

Middle cerebral artery was occluded for 24 h [35, 36].

We added the references

35. Kang JB, Shah MA, Park DJ, Koh PO. Retinoic acid regulates the ubiquitin-proteasome system in a middle cerebral artery occlusion animal model. Lab Anim Res. 2022;38: 13.

36. Kang JB, Park DJ, Shah MA, Koh PO. Retinoic acid exerts neuroprotective effects against focal cerebral ischemia by preventing apoptotic cell death. Neurosci Lett. 2021;757: 135979

3. How did the authors avoid bias in their scoring?

**Response:**

Thank you for your kind and exact comments.

Neurobehavioral deficits were assessed by the neurological deficits scoring test (Hattori et al., 2000). Neurobehavioral deficits were scored on a five-point scale according to the observation of behavioral changes as follows: no recognizable neurological deficits (0); lack of spontaneous motor activity or flexion of the contralateral forelimb (1); instinctive circling to the contralateral side (2); falling to the contralateral side or lack of spontaneous motor activity (3); dysfunctional spontaneous activity (4). As you suggest, the observation of behavioral changes can be rather subjective. Three researchers simultaneously observed behavioral changes and scored each to reduce deviations. The obtained scores were averaged and presented as the final result.

Hattori K, Lee H, Hurn PD, Crain BJ, Traystman RJ, DeVries AC. Cognitive deficits after focal cerebral ischemia in mice. Stroke. 2000;31: 1939-1944

We added sentences to explain the accuracy of the experiment

Three researchers simultaneously observed behavioral changes and scored each to reduce deviations. The obtained scores were averaged and presented as the final result.

4. The number of training sessions/day and interval of testing for corner test is missing.

**Response:**

Thank you for your kind comments.

Experimental animals were trained for accurate examination for 7 days before MCAO surgery. All animals showed a similar ratio of left turn to right turn. For accurate examination, the experimental animals were tested 10 times and the data were averaged. The experimental animals were rested for 1 minute after the test and continuously tested. The interval of the corner test was 1 minute.

We added sentences to explain the accuracy of the experiment.

We provided a corner test interval of 1 min for the accuracy of the experiment.

5. Similarly, the interval of gripping test is missing.

**Response:**

Thank you for your kind comments.

Actually, we performed five grip strength tests on the left and right forelimbs. For the accurate testing, the experimental animals were given five minutes break after the grip test. The interval of the grip strength test was five minutes.

We added sentences to improve the accuracy of the experiment.

The interval of the grip strength test was 5 min.

6. Sampling of brain sections for histopathological analysis was not adequately mentioned. Were the sections selected randomly or a systemic approach?

**Response:**

We appreciate for your kind comments.

The bregma is the anatomical point on the skull at which the coronal suture is intersected perpendicularly by the sagittal suture. For histopathological analysis, the level of brain slices was selected as 1.20 mm of bregma. We determined the level of bregma based on previously reported references (Paxinos and Watson, 2005). We previously showed infarct region from bregma +3.70 mm to bregma -2.12 mm (Shah et al., 2021). We chose the middle region of the infarct area for histopathological studies.

Paxinos G, Watson C, The rat brain in stereotaxic coordinates, seventh ed., Academic Press, San Diego, 2005.

Shah MA, Kang JB, Park DJ, Kim MO, Koh PO. Chlorogenic acid alleviates neurobehavioral disorders and brain damage in focal ischemia animal models. Neurosci Lett. 2021. 760:136085.

We added sentences to clearly describe the region of the brain tissue section.

For histopathological observation, the level of brain slices was selected as bregma 1.20 mm. The level of bregma was determined based on previously reported [40].

We added the references.

40. Paxinos G, Watson C, The rat brain in stereotaxic coordinates, seventh ed., Academic Press, San Diego, 2005.

7. There are some typos and grammar that need to be revised.

**Response:**

Thank you for your kind reviews and comments. We corrected all of the below.

Abstract

Line 30 – ‘and cerebral cortical tissues’ hanging statement.

We revised ‘and cerebral cortical tissues’ to ‘and cerebral cortical tissues were collected’

Line 31 – analysis wrongly spelled. We corrected the misspelling.

Line 38 – ‘it alleviated………” need revision.

We corrected the sentence.

Retinoic acid alleviated MCAO-induced increase of caspase-3 and cleaved caspase-3 expression.

Line 39 – tautology

We found the words in the sentence to be very similar and repetitive. Also, the content of the sentence is already mentioned in the previous sentence. Therefore, I deleted this sentence because it would be better to delete it.

Introduction

Line 48-49 – need revision.

Thank you for your comments. We revised the sentences.

The ischemic stroke is the main type of stroke, which is caused by blocked blood vessels and is also called cerebral infarction. Ischemic stroke blocks blood flow to the brain, resulting in a lack of oxygen and glucose supply [2].

Methods

Line 111 – “Animals were performed neuronal behavioral tests” animals were assessed on neurobehavioral tests.

We revised the sentence “Animals were assessed on neurobehavioral tests”

Line 123 – cm3 remove 3.

We deleted 3.

Line 137 – overnight wash with tap water – are you sure about this?

The tissue was washed with tap water overnight to remove paraformaldehyde. This process is a common method of preparing tissue for histological staining.

We modified the sentence to make it easier to understand.

Brain tissues were fixed with 4% paraformaldehyde solution and washed with tap water overnight to remove paraformaldehyde.

Duration of dehydrating and rehydrating of slides

The dehydration and rehydration steps were carried out for one minute per ethyl alcohol solution.

We added the sentence to make it easier to understand.

They were dried on slide warmer (Thermo Fisher Scientific, Waltham, MA, USA) and deparaffinized with xylene. The tissues were hydrated by gradually progressing from 100% ethyl alcohol to 70% ethyl alcohol for 1 min each, and washed with water.

Stained sections were dehydrated by gradually progressing from 70% ethyl alcohol to 100% ethyl alcohol for 1 min each. They were cleared with xylene and mounted with a permount solution (Thermo Fisher Scientific).

Line 166 – “cleared” not “cleaned”. We corrected.

Line 175 – “in ice” replace with “on ice”. We corrected.

Line 287 – remove “continuously”. We deleted “continuously”.

Discussion

Line 316 – remove “strongly”. We deleted “strongly”.

**Reviewer #2:** In this research, the author provided the data about the neuroprotective effects of retinoic acid through various neurological behavior tests including neurological deficits scoring test, corner test, and grip strength test. And explained its mechanism that retinoic acid prevented apoptosis against cerebral ischemia through phosphorylation of Akt and Bad, maintenance of phospho-Bad and 14-3-3 binding, and regulation of Bcl-2 family protein interactions. Most of the results seem likely to be valid as experimental observations.

Minor points:

Please detect the phosphorylation status of brain tissue after MCAO injury. We tried to answer your questions very carefully.

**Response:**

Thank you for your kind review.

Phosphorylation of Akt and Bad is critical for cell survival. In general, survival factors can inhibit apoptosis by activating the serine/threonine kinase Akt, which phosphorylates and inactivates components of the apoptotic machinery, including Bad and caspase 9. Actually, in the presence of survival factors, Akt phosphorylates Bad, leading to interaction with Bad and 14-3-3 proteins. Survival factor withdrawal leads to Bad dephosphorylation. Dephosphorylated Bad triggers apoptosis by inducing the expression of genes that are critical for cell death. Phosphatidylinositol 3-kinase (PI3K) induces the activation of a serine/threonine kinase called Akt or PKB. Phosphorylation of Bad by Akt inhibits Bad proapoptotic function, indicating a potent survival effect of Akt. Thus, phosphorylation of Akt and Bad is considered to be important for cell survival. We identified the phosphorylated forms of these proteins in the brain after MCAO injury. Western blot and immunohistochemical staining were performed to identify the changes in phosphorylated proteins of Akt and Bad. Moreover, total form of Akt and Bad including phosphorylated and unphosphorylated forms was detected. After MCAO injury, phosphorylated form was decreased in brain tissue and retinoic acid treatment alleviated this decrease. Western blot analyses revealed the changes in phosphorylated protein expression levels. Moreover, immunohistochemical staining revealed the localization of phosphorylated protein and the change of these proteins expression. Changes in phosphorylated proteins in brain tissues after MCAO injury were confirmed by Western blot analysis and immunohistochemical staining. We found that retinoic acid attenuates the reduction of MCAO-induced phosphorylated Akt and phosphorylated Bad. Our findings showed the changes in the phosphorylation state of brain tissue after MCAO injury. Retinoic acid contributes to the changes in phosphorylation of brain tissue during MCAO injury, mediating neuroprotective function against brain injury.

**Reviewer #3:**

1) the references cited in the past three years are not sufficient and need to be updated.

**Response:**

Thank you for your kind review. We replaced the references with the latest ones..

1. Kuriakose D, Xiao Z. Pathophysiology and treatment of stroke: present status and future perspectives. Int J Mol Sci. 2020;21: 7609.

2. He Q, Ma Y, Liu J, Zhang D, Ren J, Zhao R, et al. Biological functions and regulatory mechanisms of hypoxia-inducible factor-1α in ischemic stroke. Front Immunol. 2021;12: 801985.

3. Schädlich IS, Winzer R, Stabernack J, Tolosa E, Magnus T, Rissiek B. The role of the ATP-adenosine axis in ischemic stroke. Semin Immunopathol. 2023;45: 347-365.

4. Simats A, Liesz A. Systemic inflammation after stroke: implications for post-stroke comorbidities. EMBO Mol Med. 2022;14: e16269.

6. Dolin HH, Franco JH, Chen X, Pan ZK. Retinoic acid-induced regulation of inflammatory pathways is a potential sepsis treatment. Infect Immun. 2023;91: e0045722.

12. Ecker V, Stumpf M, Brandmeier L, Neumayer T, Pfeuffer L, Engleitner T, et al. Targeted PI3K/AKT-hyperactivation induces cell death in chronic lymphocytic leukemia. Nat Commun. 2021;12: 3526.

14. Miao R, Fang X, Wei J, Wu H, Wang X, Tian J. Akt: a potential drug target for metabolic syndrome. Front Physiol. 2022;13: 822333.

15. Levina A, Fleming KD, Burke JE, Leonard TA. Activation of the essential kinase PDK1 by phosphoinositide-driven trans-autophosphorylation. Nat Commun. 2022;13: 1874.

16. Kang JB, Shah MA, Park DJ, Koh PO. Retinoic acid regulates the ubiquitin-proteasome system in a middle cerebral artery occlusion animal model. Lab Anim Res. 2022. 38:13.

41. Gong Z, Lao D, Wu Y, Li T, Lv S, Mo X, et al. Inhibiting PI3K/Akt-signaling pathway improves neurobehavior changes in anti-NMDAR encephalitis mice by ameliorating blood-brain barrier disruption and neuronal damage. Cell Mol Neurobiol. 2023;43: 3623-3637.

2) The statistical significance in the annotations is not consistent with that in the figure and needs to be unified.

**Response:**

We appreciate for your kind comments.

We corrected the statistical significance in figure and figure legends according to your comments.

Figure 1.

**p* < 0.001, ***p* < 0.01 vs. vehicle + sham animals, #*p* < 0.001, ##*p* < 0.01 vs. vehicle + MCAO animals.

Figure 2. 3,4, 5,6.

**p* < 0.001, ***p* < 0.01 vs. vehicle + sham animals, #*p* < 0.01 vs. vehicle + MCAO animals.

3) When exploring the protective mechanism of Retinoic acid, only the phenomenon has been observed in this study. Although the results obtained were encouraging, there was no validation through the reverse way. Therefore, the results of this study are not so reliable and powerful. Therefore, it is recommended not to use words such as “demonstrated” when describing the results of this study.

**Response:**

We appreciate for your kind comments and accept your response.

We tried to exclude the word "demonstrated" and use the word such as "showed."

We revised a sentence.

Our findings showed that retinoic acid alleviates the increase of Bcl-2/Bad and Bcl-xL/Bad binding and the decrease of Bcl-2/Bax and Bcl-xL/Bax binding due to MCAO damage.
