# Supplementary material for: Retinoic acid alleviates the reduction of Akt and Bad phosphorylation and regulates Bcl-2 family protein interactions in animal models of ischemic stroke
Source: PLoS One. 2024 May 16;19(5):e0303213. doi: 10.1371/journal.pone.0303213 (PMC11098415; doi:10.1371/journal.pone.0303213)
Supplement: Attachment — Submitted filename: Dear Author.docx [file pone.0303213.s014.docx]

Dear Author,
Thanks for submitting your research manuscript entitled " Retinoic acid alleviates the reduction of Akt and Bad phosphorylation and regulates Bcl-2 family protein interactions in animal models of ischemic stroke ".

Before the final comments on this manuscript, the author needs to address the following comments in a scientific manner.
**Major concerns:-**
Please find out the following comments

- Reviewer found irrational and non-scientific justification in introduction.
- The reviewer feels author need to elaborate and justify it with latest citations with strong evidences.

**Abstract:**
- Author must nourish the Conclusion more scientifically.

**Introduction:**

- Clinical presentation of ischemic stroke patient is not well documented with clinical reference papers.

- No epidemiological data is mentioned regarding ischemic stroke (IS). Author should incorporate this data with recent citations in order to justify the rationale for this research paper.

-How would author relate the clinical manifestation of IS patient with that of experimental animal models of IS.

-Was there an adverse event or a complication being observed during the study?

**Methods:**

**-** What was the basis for the selection of only Zoletil (not other) as an anesthetic agents.

- Units of measuring behavioral parameters like Grip strength test is missing.

**Results:**

-The result is short but satisfactory.

**Discussion:**

- The concluding lines are not evident clearly, need more clarity.
- A detailed revision shortening, ordering and following the commented ideas could improve this interesting paper in a significant manner.
