# Supplementary material for: Retinoic acid alleviates the reduction of Akt and Bad phosphorylation and regulates Bcl-2 family protein interactions in animal models of ischemic stroke
Source: PLoS One. 2024 May 16;19(5):e0303213. doi: 10.1371/journal.pone.0303213 (PMC11098415; doi:10.1371/journal.pone.0303213)
Supplement: Attachment — Submitted filename: Response to reviewer.docx [file pone.0303213.s015.docx]

**Reviewer #1:**

Last comments were not addressed. I don't know if the editor did not get across to you my minor comments. The MCAO surgery protocol was written twice in the methodology section, there was 2 full stops after a sentence in line 39 and the method of election of brain slices used for CFV and TUNEL staining is still missing.

Response:

Thank you for your valuable comment and thought review for our manuscript. We removed the duplicated section. We used the brain range from bregma levels +2.0 mm to -2.0 mm for further research [Chen SH et al., 2002].

References

Chen SH, Fung PC, Cheung RT. Neuropeptide Y-Y1 receptor modulates nitric oxide level during stroke in the rat. Free Radic Biol Med. 2002;32: 776-784.

We inserted the sentence in materials and methods section.

Brain tissues from bregma levels +2.0 mm to -2.0 mm were fixed with 4% paraformaldehyde solution and washed with tap water overnight to remove paraformaldehyde. They were dehydrated with the ethyl alcohol series from 70% to 100% and washed with xylene.

**Reviewer #4:**

Thanks for your manuscript submission.

1. Clarity and Organization:

- The overall structure of the manuscript needs improvement for clarity. The introduction should provide more context on retinoic acid, ischemic stroke, and the PI3K-Akt pathway.

Response:

Thank you for your valuable comment and thought review for our manuscript. Retinoic acid has been widely studied for its regulatory mechanism in various cellular processes, including tissue damage and recovery. Retinoic acid treatment modulates the expression of key molecules involved in apoptosis such as PI3K, Akt, Bad, and caspase-3 [Jiang et al., 2018]. Retinoic acid has also been shown to enhance neural differentiation by upregulating DAX1 levels through the PI3K-Akt pathway [Nagl et al., 2009]. Ischemic stroke is a complex neurological disorder in which signaling pathways are disrupted [Wang et al., 2020]. PI3K-Akt pathway is a representative signaling pathway involved in ischemic stroke [Wang et al., 2020]. Moreover, recent study has focused on the potential therapeutic significance through PI3K-Akt pathway activation in ischemic stroke [Gu et al., 2022].

References

Jiang W, Guo M, Gong M, Chen L, Bi Y, Zhang Y, Shi Y, Qu P, Liu Y, Chen J, Li T. Vitamin A bio-modulates apoptosis via the mitochondrial pathway after hypoxic-ischemic brain damage. Mol Brain. 2018;13: 11:14.

Nagl F, Schönhofer K, Seidler B, Mages J, Allescher HD, Schmid RM, Schneider G, Saur D. Retinoic acid-induced nNOS expression depends on a novel PI3K/Akt/DAX1 pathway in human TGW-nu-I neuroblastoma cells. Am J Physiol Cell Physiol. 2009;297: C1146-56.

Wang MM, Zhang M, Feng YS, Xing Y, Tan ZX, Li WB, Dong F, Zhang F. Electroacupuncture inhibits neuronal autophagy and apoptosis via the PI3K/AKT pathway following ischemic stroke. Front Cell Neurosci. 2020;15: 14:134.

Gu C, Zhang Q, Li Y, Li R, Feng J, Chen W, Ahmed W, Soufiany I, Huang S, Long J, Chen L. The PI3K/AKT pathway-the potential key mechanisms of traditional chinese medicine for stroke. Front Med (Lausanne). 2022;31: 9:900809.

We revised and inserted the sentences the introduction part .

In previous studies, retinoic acid activated the Akt signaling pathway and promoted cell survival in various tissues [5, 33, 34]. It has also been shown to enhance neural differentiation by upregulating DAX1 levels through the PI3K-Akt pathway [35]. Ischemic stroke is a complex neurological disorder in which signaling pathways are disrupted [36]. PI3K-Akt pathway is a representative signaling pathway involved in ischemic stroke [36]. Moreover, recent study has focused on the potential therapeutic significance through PI3K-Akt pathway activation in ischemic stroke [37].

We inserted the references in reference section.

35. Nagl F, Schönhofer K, Seidler B, Mages J, Allescher HD, Schmid RM, Schneider G, Saur D. Retinoic acid-induced nNOS expression depends on a novel PI3K/Akt/DAX1 pathway in human TGW-nu-I neuroblastoma cells. Am J Physiol Cell Physiol. 2009;297: C1146-56.

36. Wang MM, Zhang M, Feng YS, Xing Y, Tan ZX, Li WB, Dong F, Zhang F. Electroacupuncture inhibits neuronal autophagy and apoptosis via the PI3K/AKT pathway following ischemic stroke. Front Cell Neurosci. 2020;15: 14:134.

37. Gu C, Zhang Q, Li Y, Li R, Feng J, Chen W, Ahmed W, Soufiany I, Huang S, Long J, Chen L. The PI3K/AKT Pathway-The Potential Key Mechanisms of Traditional Chinese Medicine for Stroke. Front Med (Lausanne). 2022;9: 900809.

- Consider providing a clearer rationale for choosing the specific doses and duration of retinoic acid administration.

Response:

We really appreciate your careful review and kind comments. We decided the dose of retinoic acid (5 mg/kg) by referring to the previously reported method [Kong et al., 2015]. The experimental animals were treated with 0.2, 1, 5, 25 mg/kg of retinoic acid to determine the protective effect of retinoic acid. Retinoic acid attenuates blood-brain barrier (BBB) permeability and infarction caused by MCAO operation. They showed that BBB-specific genes are significantly increased after treatment with either 5 or 25 mg/kg of retinoic acid compared with the vehicle group. They reported a neuroprotective effect on at least 5 mg/kg of retinoic acid. Based on the previous study, the dose of retinoic acid was determined to be 5 mg/kg, and the neuroprotective effect of retinoic acid was confirmed at this dose [Kang et al., 2021].

Kong L, Wang Y, Wang XJ, Wang XT, Zhao Y, Wang LM, et al. Retinoic acid ameliorates blood-brain barrier disruption following ischemic stroke in rats. Pharmacol Res. 2015;99: 125-136.

Kang JB, Park DJ, Shah MA, Koh PO. Retinoic acid exerts neuroprotective effects against focal cerebral ischemia by preventing apoptotic cell death. Neurosci Lett. 2021;757:135979.

We inserted the sentence in materials and methods

Animals were treated with 5 mg/kg of retinoic acid, and the dose and duration of treatment of retinoic acid were determined by the previously described method [38]. We previously confirmed the neuroprotective effect of retinoic acid at this dose and duration of retinoic acid administration [39].

We revised the references in reference list

38. Kong L, Wang Y, Wang XJ, Wang XT, Zhao Y, Wang LM, et al. Retinoic acid ameliorates blood-brain barrier disruption following ischemic stroke in rats. Pharmacol Res. 2015;99: 125-136.

293.

39. Kang JB, Shah MA, Park DJ, Koh PO. Retinoic acid regulates the ubiquitin-proteasome system in a middle cerebral artery occlusion animal model. Lab Anim Res. 2022;38: 13.

2. Background and Literature Review:

- The introduction lacks a comprehensive review of relevant literature. It should cover existing knowledge on retinoic acid, its role in stroke, and the connection with the PI3K-Akt pathway. The significance of the PI3K-Akt pathway in stroke needs more emphasis, along with recent developments in the field.

Response:

We revised and inserted the sentences the introduction part .

In previous studies, retinoic acid activated the Akt signaling pathway and promoted cell survival in various tissues [5, 33, 34]. It has also been shown to enhance neural differentiation by upregulating DAX1 levels through the PI3K-Akt pathway [35]. Ischemic stroke is a complex neurological disorder in which signaling pathways are disrupted [36]. PI3K-Akt pathway is a representative signaling pathway involved in ischemic stroke [36]. Moreover, recent study has focused on the potential therapeutic significance through PI3K-Akt pathway activation in ischemic stroke [37].

We inserted the references in reference section.

35. Nagl F, Schönhofer K, Seidler B, Mages J, Allescher HD, Schmid RM, Schneider G, Saur D. Retinoic acid-induced nNOS expression depends on a novel PI3K/Akt/DAX1 pathway in human TGW-nu-I neuroblastoma cells. Am J Physiol Cell Physiol. 2009;297: C1146-56.

36. Wang MM, Zhang M, Feng YS, Xing Y, Tan ZX, Li WB, Dong F, Zhang F. Electroacupuncture inhibits neuronal autophagy and apoptosis via the PI3K/AKT pathway following ischemic stroke. Front Cell Neurosci. 2020;15: 14:134.

37. Gu C, Zhang Q, Li Y, Li R, Feng J, Chen W, Ahmed W, Soufiany I, Huang S, Long J, Chen L. The PI3K/AKT Pathway-The Potential Key Mechanisms of Traditional Chinese Medicine for Stroke. Front Med (Lausanne). 2022;9: 900809.

3. Methodology:

- The methods section needs more details, especially regarding the experimental design, animal model characteristics, and surgical procedures.

Response:

We appreciate your careful review and kind comments. As you recommended, we inserted more details about experimental animal design and procedure.

We revised and inserted the sentences in materials and methods section.

**Experimental animal preparation**

Male Sprague Dawley (*n*=40, 210-220 g) rats were obtained from Samtako Co. (Animal Breeding Center, Osan, Korea). Male animals were used to eliminate potential confounding variables associated with sex hormones. All experimental procedures were conducted by following approved guidelines of the Institutional Animal Care and Use Committee of Gyeongsang National University (Approval number: GNU-190218-R0008). Animals were housed with controlled temperature condition with 25℃ and lighting condition with 12 h light and 12 h dark cycle. They were randomly divided into four groups as follows: vehicle + sham, retinoic acid + sham, vehicle + middle cerebral artery occlusion (MCAO), and retinoic acid + MCAO. Retinoic acid (5 mg/kg, Sigma Aldrich, St. Louis, MO, USA) was dissolved in solvent agent (polyethylene glycol, 0.9% NaCl, and ethanol; 70%/20%/10% by volume) and injected via intraperitoneal cavity four days before surgery [38]. Animals were treated with 5 mg/kg of retinoic acid, and the dose and duration of treatment of retinoic acid were determined by the previously described method [38]. We previously confirmed the neuroprotective effect of retinoic acid at this dose and duration of retinoic acid administration [39]. Vehicle group animals were injected only solvent solutions.

**Middle cerebral artery occlusion surgery**

MCAO surgery was performed to induce cerebral ischemia in the following method [40]. Animals were anesthetized by intraperitoneal injection with Zoletil (50 mg/kg, Virbac, Carros, France) 30 min after retinoic acid injection and kept in a heating pad in a supine position to maintain body temperature. Right common carotid artery (CCA) was exposed through the midline cervical incision and separated from adjacent tissues. External carotid artery (ECA) and internal carotid artery (ICA) were exposed. CCA was temporarily ligated using microvascular clamps, the laryngeal artery and cranial thyroid artery were resected, and the ECA was amputated. A 4/0 monofilament with heat-rounded tip was inserted into the cut ECA, continuously moved to the ICA until resistance was felt to block the origin of the middle cerebral artery, and ligated with ECA. Microvascular clips were removed and incised skin was sutured. Middle cerebral artery was occluded for 24 h [38, 39, 41]. Sham animals were operated the same procedure except for insertion of nylon filament. Neurobehavioral tests including neurological deficits scoring test, corner test, and grip strength test are commonly used in rodent models of stroke [42-44]. Animals were assessed on neurobehavioral tests 24 h after surgery. After anesthesia with Zoletil (Virbac), animals were quickly decapitated and sacrificed for experimentation. We tried to minimize pain to the animals and brain tissues were collected for further study.

We revised the refences.

42. Hattori K, Lee H, Hurn PD, Crain BJ, Traystman RJ, DeVries AC. Cognitive deficits after focal cerebral ischemia in mice. Stroke. 2000;31: 1939-1944.

43. Ruan J, Yao Y. Behavioral tests in rodent models of stroke. Brain Hemorrhages. 2020;1: 171-184.

44. Takeshita H, Yamamoto K, Nozato S, Inagaki T, Tsuchimochi H, Shirai M, et al. Modified forelimb grip strength test detects aging-associated physiological decline in skeletal muscle function in male mice. Sci Rep. 2017;7: 42323.

- Specify the criteria for selecting the 5 mg/kg dose of retinoic acid and the rationale for the four-day treatment period.

Response:

We appreciate your careful review and kind comments. We decided the dose of retinoic acid (5 mg/kg) by referring to the previously reported method [Kong et al., 2015]. The experimental animals were treated with 0.2, 1, 5, 25 mg/kg of retinoic acid to determine the protective effect of retinoic acid. Retinoic acid attenuates blood-brain barrier (BBB) permeability and infarction caused by MCAO operation. They showed that BBB-specific genes are significantly increased after treatment with either 5 or 25 mg/kg of retinoic acid compared with the vehicle group. They reported a neuroprotective effect on at least 5 mg/kg of retinoic acid. They also continuously treated retinoic acid 4 days before MCAO surgery, so we tried their method by referring to Kong et al. Actually, 5 mg/kg of retinoic acid was continuously administered to the experimental animals for 4 days before MCAO surgery, and we clearly identified the neuroprotective effect of retinoic acid at this dose and duration of retinoic acids [Kang et al., 2021].

References

Kong L, Wang Y, Wang XJ, Wang XT, Zhao Y, Wang LM, Chen ZY Retinoic acid ameliorates blood-brain barrier disruption following ischemic stroke in rats. Pharmacol Res. 2015;99: 125-136.

Kang JB, Park DJ, Shah MA, Koh PO. Retinoic acid exerts neuroprotective effects against focal cerebral ischemia by preventing apoptotic cell death. Neurosci Lett. 2021;757:135979.

We modified and inserted the sentence in materials and methods

Animals were treated with 5 mg/kg of retinoic acid, and the dose and duration of treatment of retinoic acid were determined by the previously described method [38]. We previously confirmed the neuroprotective effect of retinoic acid at this dose and duration of retinoic acid administration [39].

- Provide information on the choice of neurobehavioral tests used and justify why these specific tests were selected.

Response:

Thank you for your kind review and comment. The neurological deficit score test, corner test, and grip strength test are motor/sensory motor and cognitive tests commonly used in rodent models of stroke [Ruan et al., 2020]. Neurological deficits scoring test is a combined assessment method that observes various neurological functions such as balance, coordination, reflexes, and sensory perception. The corner test observes a turning pattern towards the strong side during the corner turn. The grip strength test assesses motor function by measuring force. Behavioral tests were performed to confirm the behavioral deficits induced by MCAO and to investigate the effects of retinoic acid administration on behavioral improvement and neuroprotection. Neurological deficit score tests showed that retinoic acid-treated animals with MCAO damage scored lower than vehicle-treated animals with MCAO damage. In the corner test, retinoic acid-treated animals with MCAO damage showed a lower frequency of rotation in the same direction as the lesion, compared with vehicle-treated animal with MCAO damage. In the grip test, retinoic acid-treated animals with MCAO damage had a higher grip strength on the contralateral side compared to vehicle-treated animal with MCAO damage. These results confirmed that MCAO induces behavioral deficits and retinoic acid administration improves behavioral deficits. Therefore, we can demonstrate that retinoic acid has neuroprotective effects in focal cerebral ischemia.

References

Ruan J, Yao Y. Behavioral tests in rodent models of stroke. Brain Hemorrhages. 2020;1: 171-184.

We inserted the sentence in materials and methods.

Neurobehavioral tests including neurological deficits scoring test, corner test, and grip strength test are commonly used in rodent models of stroke [42-44]. Animals were assessed on neurobehavioral tests 24 h after surgery.

We inserted the reference in reference section

42. Ruan J, Yao Y. Behavioral tests in rodent models of stroke. Brain Hemorrhages. 2020;1: 171-184.

43. Hattori K, Lee H, Hurn PD, Crain BJ, Traystman RJ, DeVries AC. Cognitive deficits after focal cerebral ischemia in mice. Stroke. 2000;31: 1939-1944.

44. Takeshita H, Yamamoto K, Nozato S, Inagaki T, Tsuchimochi H, Shirai M, et al. Modified forelimb grip strength test detects aging-associated physiological decline in skeletal muscle function in male mice. Sci Rep. 2017;7: 42323.

4. Results:

- The results section lacks clarity in presenting the findings. Consider reorganizing and presenting the results in a more logical sequence.

Response:

Thank you for your thoughtful comment. Various experiments were performed to confirm the regulation of phospho-AKT and phospho-Bad in ischemic stroke by retinoic acid treatment. Behavioral tests such as neurological deficit score, corner test, and grip strength test were performed to confirm the neuroprotective function of retinoic acid in ischemic stroke animals. In addition, the results of cresyl violet staining and TUNEL staining showed that retinoic acid administration reduced the damage of cerebral cortex in MCAO animal. Western blot analysis and immunohistochemistry staining were used to confirm the changes of expression of phospho-AKT and phospho-Bad by retinoic acid in ischemic stroke. We showed that retinoic acid administration alleviates the reduction of 14-3-3 in MACO animals. We also confirmed the changes in 14-3-3/phospho-Bad, Bcl-2/Bad, Bcl-xL/Bad, Bcl-2/Bax, Bcl-xL/Bax by retinoic acid in ischemic stroke using immunoprecipitation analysis. The binding of these proteins acts as a very important factor in carrying out the neuroprotective effects of retinoic acid in ischemic stroke. Finally, the changes of expression of caspase-3 and cleaved caspase-3 were observed using Western blot analysis. We tried to organize the contents of the experimental results systematically according to the experimental process. We think the arrangement of the results is relatively logical. If we reorganize the results, then the descriptive and discussion parts of the results must also be rearranged. Moreover, our logical explanation must also be rearranged. Unfortunately, it is difficult to rearrange the results in the current situation. We are sorry about this and ask for your generous understanding.

- Include statistical analyses and significance levels for the observed changes.

Response:

Thank you for your thoughtful comment. We included the p-values using, * / ** / # / ##. * and # indicate statistical significance at the p<0.001 level for vehicle + sham and vehicle + MCAO animals, respectively. ** and ## indicate statistical significance at the p<0.01 level for vehicle + sham and vehicle + MCAO animals, respectively.

We mentioned in figure legend

**p* < 0.001, ***p* < 0.01 vs. vehicle + sham animals, #*p* < 0.001, ##*p* < 0.01 vs. vehicle + MCAO animals

- Clarify the timeline of events and measurements, especially in relation to the administration of retinoic acid and the induction of ischemic stroke.

Response:

We appreciate your careful review and kind comments. We decided the dose of retinoic acid (5 mg/kg) by referring to the previously reported method [Kong et al., 2015]. The experimental animals were treated with 0.2, 1, 5, 25 mg/kg of retinoic acid to determine the protective effect of retinoic acid. Retinoic acid attenuates blood-brain barrier (BBB) permeability and infarction caused by MCAO operation. They showed that BBB-specific genes are significantly increased after treatment with either 5 or 25 mg/kg of retinoic acid compared with the vehicle group. They reported a neuroprotective effect on at least 5 mg/kg of retinoic acid. They also continuously treated retinoic acid 4 days before MCAO surgery, so we tried their method by referring to Kong et al. Actually, 5 mg/kg of retinoic acid was continuously administered to the experimental animals for 4 days before MCAO surgery, and we clearly identified the neuroprotective effect of retinoic acid at this dose and duration of retinoic acids (Kang et al., 2021).

References

Kong L, Wang Y, Wang XJ, Wang XT, Zhao Y, Wang LM, Chen ZY Retinoic acid ameliorates blood-brain barrier disruption following ischemic stroke in rats. Pharmacol Res. 2015;99: 125-136.

Kang JB, Park DJ, Shah MA, Koh PO. Retinoic acid exerts neuroprotective effects against focal cerebral ischemia by preventing apoptotic cell death. Neurosci Lett. 2021;757:135979.

We modified and inserted the sentence in materials and methods

Retinoic acid (5 mg/kg, Sigma Aldrich, St. Louis, MO, USA) was dissolved in solvent agent (polyethylene glycol, 0.9% NaCl, and ethanol; 70%/20%/10% by volume) and injected via intraperitoneal cavity four days before surgery. Animals were treated with 5 mg/kg of retinoic acid, and the dose and duration of treatment of retinoic acid were determined by the previously described method [38]. We previously confirmed the neuroprotective effect of retinoic acid at this dose and duration of retinoic acid administration [39]. Vehicle group animals were injected only solvent solutions.

5. Discussion:

- The discussion needs to be more thorough and should interpret the results in the context of existing literature.

Response:

Thank you for your comment. As you recommended, we revised the discussion section more interpretive with result.

This study supported the neuroprotective effects of retinoic acid through various neurological behavior tests including neurological deficits scoring test, corner test, and grip strength test. Surgical MCAO was performed in the right brain of rats. A reduction of grip strength was observed in the left forelimb. These results showed neurological behavioral deficits in the contralateral side of the damaged brain. Retinoic acid mitigated the neurological disorder caused by MCAO and exerted neuroprotective effects. Furthermore, the morphological changes were confirmed in cresyl violet-stained tissues. Retinoic acid treatment decreased infarct areas caused by MCAO damage and reduced the number of damaged cells. We recently reported that retinoic acid alleviates the increases in brain edema and infarction volume due to MCAO damage [41]. The present study also confirmed that retinoic acid attenuates the histopathological changes and increase in the number of TUNEL-positive cells in the cerebral cortex with MCAO. Stroke is a complex disease involving a variety of mechanisms. The PI3K-Akt pathway is a representative pathway for neuronal survival and protection. PI3K-Akt is a key mediator of cerebral ischemic stroke [37]. The present study focused on the neuroprotective effects of retinoic acid by modulating the PI3K-Akt signaling pathway. This study additionally elucidated that retinoic acid performs neuroprotective functions through activation of the PI3K-Akt-Bad signaling pathway and regulation of Bcl-2 family protein interactions in a stroke animal model.

Previous studies have demonstrated the efficacy of retinoic acid in various diseases [55, 56]. Especially in the brain, retinoic acid has neuroprotective effects in neurodegenerative diseases such as amyotrophic lateral sclerosis, Parkinson's and ischemic stroke. In this study, we focused on the specific protective mechanism of retinoic acid on cerebral ischemia [57-59]. The PI3K-Akt pathway has shown therapeutic and preventive effects in various neurological disorders including stroke, Alzheimer’s disease, and Parkinson’s disease [60, 61]. Therefore, it is hypothesized that the regulation of the PI3K-Akt pathway by retinoic acid treatment may also be involved in the prevention and treatment of stroke. We previously showed a decrease in phospho-Bad due to MCAO damage and further described the mitigation of phospho-Bad decrease caused by retinoic acid. The changes in phospho-Bad expression due to retinoic acid treatment in MCAO indicate the importance of phospho-Bad in neuroprotective effects of retinoic acid. The maintenance of phospho-Akt and phospho-Bad plays a crucial role in cell survival. Thus, the results show that the PI3K/Akt signaling pathway contributes to the neuroprotective mechanism of retinoic acid in cerebral ischemia.

- Provide a detailed explanation of how retinoic acid affects the PI3K-Akt pathway and its downstream proteins.

Response:

Thank you for your valuable comment. Retinoic acid has been widely studied for its regulatory role in various cellular processes, including tissue damage and recovery. Retinoic acid treatment modulates the expression of molecules involved in cell signaling, such as PI3K, Akt, Bad, caspase-3, caspase-8, Bcl-2, Bax, and Bid [Jiang et al., 2018]. It directly interacts with cell membrane receptors such as the retinoic acid receptor, leading to the activation of PI3K pathway [Wu et al., 2024]. Activation of PI3K induces phosphorylation of Akt and regulates downstream proteins such as Bad, thereby promoting cell survival and proliferation [Qin C et al., 2022]. Our finding showed that retinoic acid regulates expressions of PI3K, Akt, Bad and modulates Bcl-2 family protein in ischemic brain damage. We suggest that retinoic acid protects brain tissues against cerebral ischemic damage by regulating PI3K-Akt-Bad pathway and modulating Bcl-2 family proteins.

References

Jiang W, Guo M, Gong M, Chen L, Bi Y, Zhang Y, Shi Y, Qu P, Liu Y, Chen J, Li T. Vitamin A bio-modulates apoptosis via the mitochondrial pathway after hypoxic-ischemic brain damage. Mol Brain. 2018;11: 14.

Wu D, Khan FA, Zhang K, Pandupuspitasari NS, Negara W, Guan K, Sun F, Huang C. Retinoic acid signaling in development and differentiation commitment and its regulatory topology. Chem Biol Interact. 2024;387: 110773.

Qin C, Yang S, Chu YH, Zhang H, Pang XW, Chen L, Zhou LQ, Chen M, Tian DS, Wang W. Signaling pathways involved in ischemic stroke: molecular mechanisms and therapeutic interventions. Signal Transduct Target Ther. 2022;7: 215.

- Discuss potential limitations of the study and avenues for future research.

Response:

Thank you for your valuable comment.

We found that the PI3K-Akt-Bad signaling pathway is involved in the neuroprotective mechanism of retinoic acid in stroke models. However, further research is needed on other signaling pathways such as Akt-FKHR and Akt-GSK 3 beta is involved in stroke models. We also need to find more factors that are regulated by retinoic acid in the ischemic stroke. Further studies should be performed that provide therapeutic effects of retinoic acid in stoke model.

We revised the discussion section.

We also need to find more factors that are regulated by retinoic acid in the ischemic stroke. Further studies should be performed that provide therapeutic effects of retinoic acid in stoke model.

- Address the clinical relevance of the findings and potential implications for stroke therapy.

Response:

Thank you for your kind comments. Many studies have focused on the PI3K-Akt signaling pathway as a target for neurovegetative diseases including stroke, Alzheimer’s disease, and Parkinson’s disease [Long et al., 2021; Goyal et al., 2023]. Moreover, retinoic acid has neuroprotective effects in neurodegenerative diseases such as amyotrophic lateral sclerosis, Parkinson's disease and ischemic stroke [Zhu et al., 2020; Esteves et al., 2015; Cai et al., 2019]. This means that retinoic acid has already passed human safety. Therefore, targeting the PI3K-Akt signaling pathway using retinoic acid may be helpful in the development of ischemic stroke treatment.

Long HZ, Cheng Y, Zhou ZW, Luo HY, Wen DD, Gao LC. PI3K/AKT signal pathway: A target of natural products in the prevention and treatment of Alzheimer's disease and Parkinson's disease. Front Pharmacol. 2021;12: 648636.

Goyal A, Agrawal A, Verma A, Dubey N. The PI3K-AKT pathway: A plausible therapeutic target in Parkinson's disease. Exp Mol Pathol. 2023;129: 104846.

Zhu Y, Liu Y, Yang F, Chen W, Jiang J, He P, et al. All-trans retinoic acid exerts neuroprotective effects in amyotrophic lateral sclerosis-like Tg (SOD1*G93A)1Gur mice. Mol Neurobiol. 2020;57(8):3603-3615.

Esteves M, Cristóvão AC, Saraiva T, Rocha SM, Baltazar G, Ferreira L, et al. Retinoic acid-loaded polymeric nanoparticles induce neuroprotection in a mouse model for Parkinson's disease. Front Aging Neurosci. 2015;7:20.

Cai W, Wang J, Hu M, Chen X, Lu Z, Bellanti JA, et al. All trans-retinoic acid protects against acute ischemic stroke by modulating neutrophil functions through STAT1 signaling. J Neuroinflammation. 2019;16(1):175.

We inserted the sentence in discussion section.

Previous studies have demonstrated the efficacy of retinoic acid in various diseases [55, 56]. Especially in the brain, retinoic acid has neuroprotective effects in neurodegenerative diseases such as amyotrophic lateral sclerosis, Parkinson's and ischemic stroke. In this study, we focused on the specific protective mechanism of retinoic acid on cerebral ischemia [57-59]. The PI3K-Akt pathway has shown therapeutic and preventive effects in various neurological disorders including stroke, Alzheimer’s disease, and Parkinson’s disease [60, 61]. Therefore, it is hypothesized that the regulation of the PI3K-Akt pathway by retinoic acid treatment may also be involved in the prevention and treatment of stroke.

55. Caricasulo MA, Zanetti A, Terao M, Garattini E, Paroni G. Cellular and micro-environmental responses influencing the antitumor activity of all-trans retinoic acid in breast cancer. Cell Commun Signal. 2024;22: 127.

56. Al-Marsoummi S, Mehus AA, Garrett SH, Sens DA, Somji S. The Effect of Retinoic Acid on Arsenite-Transformed Malignant UROtsa Bladder Cancer Cells: In Vitro Model of Basal Muscle-Invasive Bladder Cancer. Cancers (Basel). 2024;16: 1178.

57. Zhu Y, Liu Y, Yang F, Chen W, Jiang J, He P, et al. All-trans retinoic acid exerts neuroprotective effects in amyotrophic lateral sclerosis-like Tg (SOD1*G93A)1Gur mice. Mol Neurobiol. 2020;57(8):3603-3615.

58. Esteves M, Cristóvão AC, Saraiva T, Rocha SM, Baltazar G, Ferreira L, et al. Retinoic acid-loaded polymeric nanoparticles induce neuroprotection in a mouse model for Parkinson's disease. Front Aging Neurosci. 2015;7:20.

59. Cai W, Wang J, Hu M, Chen X, Lu Z, Bellanti JA, et al. All trans-retinoic acid protects against acute ischemic stroke by modulating neutrophil functions through STAT1 signaling. J Neuroinflammation. 2019;16(1):175.

60. Long HZ, Cheng Y, Zhou ZW, Luo HY, Wen DD, Gao LC. PI3K/AKT signal pathway: A target of natural products in the prevention and treatment of Alzheimer's disease and Parkinson's disease. Front Pharmacol. 2021;12: 648636.

61. Goyal A, Agrawal A, Verma A, Dubey N. The PI3K-AKT pathway: A plausible therapeutic target in Parkinson's disease. Exp Mol Pathol. 2023;129: 104846.

6. Figures and Tables:

- Ensure that figures and tables are clear and easy to interpret. Include proper labeling and legends for all figures and tables. Consider whether additional figures or tables could enhance the presentation of results.

Response:

Thank you for your thoughtful comment. We included the p-values using, * / ** / # / ##. * and # indicate statistical significance at the p<0.001 level for vehicle + sham and vehicle + MCAO animals, respectively. ** and ## indicate statistical significance at the p<0.01 level for vehicle + sham and vehicle + MCAO animals, respectively.

We mentioned in figure legend

**p* < 0.001, ***p* < 0.01 vs. vehicle + sham animals, #*p* < 0.001, ##*p* < 0.01 vs. vehicle + MCAO animals

Thank you for your thoughtful comment. Various experiments were performed to confirm the regulation of phospho-AKT and phospho-Bad in ischemic stroke by retinoic acid treatment. Behavioral tests such as neurological deficit score, corner test, and grip strength test were performed to confirm the neuroprotective function of retinoic acid in ischemic stroke animals. In addition, the results of cresyl violet staining and TUNEL staining showed that retinoic acid administration reduced the damage of cerebral cortex in MCAO animal. Western blot analysis and immunohistochemistry staining were used to confirm the changes of expression of phospho-AKT and phospho-Bad by retinoic acid in ischemic stroke. We showed that retinoic acid administration alleviates the reduction of 14-3-3 in MACO animals. We also confirmed the changes in 14-3-3/phospho-Bad, Bcl-2/Bad, Bcl-xL/Bad, Bcl-2/Bax, Bcl-xL/Bax by retinoic acid in ischemic stroke using immunoprecipitation analysis. The binding of these proteins acts as a very important factor in carrying out the neuroprotective effects of retinoic acid in ischemic stroke. Finally, the changes of expression of caspase-3 and cleaved caspase-3 were observed using Western blot analysis. We tried to organize the contents of the experimental results systematically according to the experimental process. We think the arrangement of the results is relatively logical. If we reorganize the results, then the descriptive and discussion parts of the results must also be rearranged. Moreover, our logical explanation must also be rearranged. Unfortunately, it is difficult to rearrange the results in the current situation. We are sorry about this and ask for your generous understanding.

7. Language and Style:

- Improve the overall writing style for clarity and precision. Check grammar and sentence structure throughout the manuscript. Eliminate unnecessary repetition and jargon that may hinder understanding for readers outside the specific field.

Response:

Thank you for your kind consideration. We corrected the grammatical errors. We have completed the correction from the English proofreading company.

8. Conclusion:

- The conclusion should be strengthened, summarizing the key findings and their implications more clearly. Avoid introducing new information in the conclusion; focus on summarizing and emphasizing the main contributions of the study.

Response:

Thank you for your comment. As you recommended, we revised the conclusion section.

We revised the conclusion section.

Taken together, our results demonstrated that retinoic acid treatment induces the phosphorylation of Akt and Bad, regulates the interaction with Bcl-2 family proteins, and modulates the expression of caspase-3 in stroke animal models. Retinoic acid inhibits the process of apoptosis, promotes neuronal survival, and ultimately exerts neuroprotective effects in ischemic stroke. In conclusion, retinoic acid provides neuroprotective effects through mitigation of Akt and Bad phosphorylation and modulation of Bcl-2 family protein interactions in ischemic stroke.

**Reviewer #5:**

Before the final comments on this manuscript, the author needs to address the comments in a scientific manner.

Response:

Thank you for your kind and thoughtful comment. We revised the manuscript more scientifically.

We revised the introduction section

In previous studies, retinoic acid activated the Akt signaling pathway and promoted cell survival in various tissues [5, 33, 34]. Retinoic acid has been shown to enhance neural differentiation by upregulating DAX1 levels via the PI3K-Akt pathway [35]. Ischemic stroke is a complex neurological disorder, and the PI3K-Akt pathway is also involved in this process [36]. Moreover, recent research has demonstrated the potential therapeutic significance of alleviating PI3K-Akt pathway dysfunction in ischemic stroke [37].

We revised the conclusion section.

Taken together, our results demonstrated that retinoic acid treatment induces the phosphorylation of Akt and Bad, regulates the interaction with Bcl-2 family proteins, and modulates the expression of caspase-3 in stroke animal models. Retinoic acid inhibits the process of apoptosis, promotes neuronal survival, and ultimately exerts neuroprotective effects in ischemic stroke. In conclusion, retinoic acid provides neuroprotective effects through mitigation of Akt and Bad phosphorylation and modulation of Bcl-2 family protein interactions in ischemic stroke.

We inserted the references in reference section.

35. Nagl F, Schönhofer K, Seidler B, Mages J, Allescher HD, Schmid RM, Schneider G, Saur D. Retinoic acid-induced nNOS expression depends on a novel PI3K/Akt/DAX1 pathway in human TGW-nu-I neuroblastoma cells. Am J Physiol Cell Physiol. 2009;297: C1146-56.

36. Wang MM, Zhang M, Feng YS, Xing Y, Tan ZX, Li WB, Dong F, Zhang F. Electroacupuncture inhibits neuronal autophagy and apoptosis via the PI3K/AKT pathway following ischemic stroke. Front Cell Neurosci. 2020;15: 14:134.

37. Gu C, Zhang Q, Li Y, Li R, Feng J, Chen W, Ahmed W, Soufiany I, Huang S, Long J, Chen L. The PI3K/AKT pathway-the potential key mechanisms of traditional chinese medicine for stroke. Front Med. 2022;31: 9:900809.

**Reviewer #6:**

The paper written by Ju-Bin Kang et al. found that retinoic acid can effectively relieve the brain nerve injury caused by ischemic stroke. Overall, the study looks very interesting, but there are still some questions that need to be addressed:

1.Why were male rats chosen? Is there gender difference for ischemic stroke?

Response:

We sincerely appreciate your thorough review. The decision to use male rats in our study stems from several considerations regarding the pathophysiology of ischemic stroke. Gender differences have been noted in the various pathologic mechanisms underlying stroke [Gibson et al., 2013]. In particular, female rats have an estrous cycle, and sex hormones such as estrogen and progesterone are severely changed according to the estrous cycle [Huang et al., 2022]. Estrogen is known to protect nerve cells, inhibit apoptosis, and participate in the survival of nerve cells. In addition, neuroprotective effects of estrogens in focal cerebral ischemia was reported [Zhong et al., 2023]. When female animals are used in the experiment, an ovariectomy have to perform to eliminate female sex hormones, which is to exclude the effect of female sex hormones. We used male animals to exclude changes in sex hormones that change with the estrous cycle in females. In conclusion, we used male animals to eliminate potential confounding factors associated with sex hormones, thereby providing clear insight into the specific mechanisms of ischemic stroke and its therapeutic response.

References

Huang S, Liu L, Tang X, Xie S, Li X, Kang X, Zhu S. Research progress on the role of hormones in ischemic stroke. Front Immunol. 2022;13: 1062977.

Zhong X, Sun Y, Lu Y, Xu L. Immunomodulatory role of estrogen in ischemic stroke: neuroinflammation and effect of sex. Front Immunol. 2023;14: 1164258.

We inserted the sentence in materials and methods section

Male Sprague Dawley (*n*=40, 210-220 g) rats were obtained from Samtako Co. (Animal Breeding Center, Osan, Korea). Male animals were used to eliminate potential confounding factors associated with sex hormones.

2. Why was retinoic acid injected four days before surgery?

Response:

We appreciate your careful review and kind comments. We determined the day of treatment in experimental animals by referring to the previously reported method [Kong et al., 2015]. They treated retinoic acid consecutively 4 days before MCAO surgery. They demonstrated that retinoic acid reduced blood-brain barrier disruption and alleviated cerebral infarction caused by MCAO surgery. According to previously reported method, 5 mg/kg of retinoic acid was continuously administered to experimental animals for 4 days prior to MCAO surgery. Moreover, we previously confirmed the neuroprotective effect of retinoic acid at this dose and duration of retinoic acid administration [Kang et al., 2021]. We have obtained clear results on the neuroprotective effects of retinoic acid in ischemic stroke. Thus, we chose to inject retinoic acid four days before surgery.

Kong L, Wang Y, Wang XJ, Wang XT, Zhao Y, Wang LM, et al. Retinoic acid ameliorates blood-brain barrier disruption following ischemic stroke in rats. Pharmacol Res. 2015;99: 125-136.

Kang JB, Park DJ, Shah MA, Koh PO. Retinoic acid exerts neuroprotective effects against focal cerebral ischemia by preventing apoptotic cell death. Neurosci Lett. 2021;757:135979.

We inserted the sentence in materials and methods

Animals were treated with 5 mg/kg of retinoic acid, and the dose and duration of treatment of retinoic acid were determined by the previously described method [33]. We previously confirmed the neuroprotective effect of retinoic acid at this dose and duration of retinoic acid administration [39].

3. Besides PI3K-Akt signaling pathway, what other pathways are involved in ischemic stroke?

Response:

Thank you for your valuable comment. It is known that many signaling pathways are related with ischemic stroke. Death-associated protein kinase 1 (DAPK1) is a Ca2+/calmodulin-dependent serine/threonine-protein kinase, whose phosphorylation contributes to apoptosis. DAPK1 participates in excitotoxicity in ischemic stroke [Noori et al., 2022]. Synaptic NMDAR activation and Ca2+ influx activate the ERK signaling pathway and nuclear Ca2+/calmodulin-dependent protein kinases, which in turn phosphorylate and activate CREB [Impey et al., 2002]. Although many pathways are associated with ischemic stroke, PI3K-Akt is a major mediator of cerebral ischemia [Gu et al., 2022]. The PI3K-AKT signaling pathway regulates calcium homeostasis by regulating excitotoxicity-related signaling pathways [George and Steinberg, 2015]. Moreover, PI3K-Akt signaling pathway is related with synaptic NMDARs. Stroke is a complex disease involving a variety of mechanisms. The PI3K-Akt pathway is a representative pathway for neuronal survival and protection. Therefore, the present study focused on the neuroprotective effects of retinoic acid by modulating the PI3K-Akt signaling pathway.

Noori T, Shirooie S, Sureda A, Sobarzo-Sanchez E, Dehpour AR, Saldías M, Akkol EK. Regulation of DAPK1 by natural products: an important target in treatment of stroke. Neurochem Res. 2022;47: 2142-2157.

Impey S, Fong AL, Wang Y, Cardinaux JR, Fass DM, Obrietan K, Wayman GA, Storm DR, Soderling TR, Goodman RH. Phosphorylation of CBP mediates transcriptional activation by neural activity and CaM kinase IV. Neuron. 2002;34: 235-44.

George PM, Steinberg GK. Novel stroke therapeutics: unraveling stroke pathophysiology and its impact on clinical treatments. Neuron. 2015;87: 297-309.

Gu C, Zhang Q, Li Y, Li R, Feng J, Chen W, Ahmed W, Soufiany I, Huang S, Long J, Chen L. The PI3K/AKT pathway-the potential key mechanisms of traditional chinese medicine for stroke. Front Med (Lausanne). 2022;9: 900809.

We inserted the sentence in discussion section.

Stroke is a complex disease involving a variety of mechanisms. The PI3K-Akt pathway is a representative pathway for neuronal survival and protection. PI3K-Akt is a key mediator of cerebral ischemic stroke [37]. The present study focused on the neuroprotective effects of retinoic acid by modulating the PI3K-Akt signaling pathway. This study additionally elucidated that retinoic acid performs neuroprotective functions through activation of the PI3K-Akt-Bad signaling pathway and regulation of Bcl-2 family protein interactions in a stroke animal model.

We inserted the reference.

37. Gu C, Zhang Q, Li Y, Li R, Feng J, Chen W, Ahmed W, Soufiany I, Huang S, Long J, Chen L. The PI3K/AKT pathway-the potential key mechanisms of traditional chinese medicine for stroke. Front Med (Lausanne). 2022;9: 900809.

4. How to understand that the grip strength of the left and right forelimbs was examined, but only one forelimb is used in each test?

Response:

Thank you for your comment.

Cerebral ischemic stroke causes muscle imbalance. Grip strength tests were performed on each forelimb to determine muscle strength by retinoic acid administration in stroke. Animals are naturally inclined to use both forelimbs for grasping. We measured grip strength for each of the forelimbs, which provides a more consistent measurement. The test was performed with only one forelimb at a time. For example, when examining right forelimb, the other left forelimb was wrapped with masking tape.

We inserted the sentence in materials and methods

The test was performed with only one forelimb at a time. For example, when examining right forelimb, the other left forelimb was wrapped with masking tape.

5. Are there any references for supporting the content in Lines 57-59?

Response:

Thank you for your comment. As you recommended, we insert the references for supporting the sentences.

We revised the introduction section.

However, in the absence of growth or survival factors, Bad interacts with Bcl-2 or Bcl-xL and dissociates Bax from the Bcl-2/Bax and Bcl-xL/Bax complexes [22]. Activated Bax releases cytochrome c from mitochondria to the cytosol and continuously activates the caspase cascade, which results in apoptotic cell death [23].

We inserted the references

22. Garenne D, Renault TT, Manon S. Bax mitochondrial relocation is linked to its phosphorylation and its interaction with Bcl-xL. Microb Cell. 2016;3: 597-605.

23. Garrido C, Galluzzi L, Brunet M, Puig PE, Didelot C, Kroemer G. Mechanisms of cytochrome c release from mitochondria. Cell Death Differ. 2006;13: 1423-33.

6.There are some grammatical errors in this manuscript, and it is better to find a native speaker to correct it.

Response:

Thank you for your kind consideration. We corrected the grammatical errors. We have completed the correction from the English proofreading company.

7. In the Reference, please check them carefully and modify them according to the requirements of the Journal.

Response:

Thank you for your kind comment. As you recommended, we checked the references. Also, we added more references to elaborate on the paper.

22. Garenne D, Renault TT, Manon S. Bax mitochondrial relocation is linked to its phosphorylation and its interaction with Bcl-xL. Microb Cell. 2016;3: 597-605.

23. Garrido C, Galluzzi L, Brunet M, Puig PE, Didelot C, Kroemer G. Mechanisms of cytochrome c release from mitochondria. Cell Death Differ. 2006;13: 1423-33.

35. Nagl F, Schönhofer K, Seidler B, Mages J, Allescher HD, Schmid RM, Schneider G, Saur D. Retinoic acid-induced nNOS expression depends on a novel PI3K/Akt/DAX1 pathway in human TGW-nu-I neuroblastoma cells. Am J Physiol Cell Physiol. 2009;297: C1146-56.

36. Wang MM, Zhang M, Feng YS, Xing Y, Tan ZX, Li WB, Dong F, Zhang F. Electroacupuncture inhibits neuronal autophagy and apoptosis via the PI3K/AKT pathway following ischemic stroke. Front Cell Neurosci. 2020;15: 14:134.

37. Gu C, Zhang Q, Li Y, Li R, Feng J, Chen W, Ahmed W, Soufiany I, Huang S, Long J, Chen L. The PI3K/AKT pathway-the potential key mechanisms of traditional chinese medicine for stroke. Front Med. 2022;31: 9:900809.

42. Ruan J, Yao Y. Behavioral tests in rodent models of stroke. Brain Hemorrhages. 2020;1: 171-184.

55. Caricasulo MA, Zanetti A, Terao M, Garattini E, Paroni G. Cellular and micro-environmental responses influencing the antitumor activity of all-trans retinoic acid in breast cancer. Cell Commun Signal. 2024;22: 127.

56. Al-Marsoummi S, Mehus AA, Garrett SH, Sens DA, Somji S. The Effect of Retinoic Acid on Arsenite-Transformed Malignant UROtsa Bladder Cancer Cells: In Vitro Model of Basal Muscle-Invasive Bladder Cancer. Cancers (Basel). 2024;16: 1178.

57. Zhu Y, Liu Y, Yang F, Chen W, Jiang J, He P, et al. All-trans retinoic acid exerts neuroprotective effects in amyotrophic lateral sclerosis-like Tg (SOD1*G93A)1Gur mice. Mol Neurobiol. 2020;57(8):3603-3615.

58. Esteves M, Cristóvão AC, Saraiva T, Rocha SM, Baltazar G, Ferreira L, et al. Retinoic acid-loaded polymeric nanoparticles induce neuroprotection in a mouse model for Parkinson's disease. Front Aging Neurosci. 2015;7:20.

59. Cai W, Wang J, Hu M, Chen X, Lu Z, Bellanti JA, et al. All trans-retinoic acid protects against acute ischemic stroke by modulating neutrophil functions through STAT1 signaling. J Neuroinflammation. 2019;16(1):175.

60. Long HZ, Cheng Y, Zhou ZW, Luo HY, Wen DD, Gao LC. PI3K/AKT signal pathway: A target of natural products in the prevention and treatment of Alzheimer's disease and Parkinson's disease. Front Pharmacol. 2021;12: 648636.

61. Goyal A, Agrawal A, Verma A, Dubey N. The PI3K-AKT pathway: A plausible therapeutic target in Parkinson's disease. Exp Mol Pathol. 2023;129: 104846.
